# Rational optimization of *tolC* as a powerful dual selectable marker for genome engineering

**DOI:** 10.1093/nar/gkt1374

**Published:** 2014-01-22

**Authors:** Christopher J. Gregg, Marc J. Lajoie, Michael G. Napolitano, Joshua A. Mosberg, Daniel B. Goodman, John Aach, Farren J. Isaacs, George M. Church

**Affiliations:** ^1^Department of Genetics and Wyss Institute for Biologically Inspired Engineering, Harvard Medical School, Boston, MA 02115, USA, ^2^Program in Chemical Biology, Harvard University, Cambridge, MA 02138, USA, ^3^Biological and Biomedical Sciences, Harvard Medical School, Boston, MA 02115, USA and ^4^Molecular, Cellular, Developmental and Systems Biology Institute, Yale University, New Haven, CT 06516, USA

## Abstract

Selection has been invaluable for genetic manipulation, although counter-selection has historically exhibited limited robustness and convenience. TolC, an outer membrane pore involved in transmembrane transport in *E. coli*, has been implemented as a selectable/counter-selectable marker, but counter-selection escape frequency using colicin E1 precludes using *tolC* for inefficient genetic manipulations and/or with large libraries. Here, we leveraged unbiased deep sequencing of 96 independent lineages exhibiting counter-selection escape to identify loss-of-function mutations, which offered mechanistic insight and guided strain engineering to reduce counter-selection escape frequency by ∼40-fold. We fundamentally improved the *tolC* counter-selection by supplementing a second agent, vancomycin, which reduces counter-selection escape by 425-fold, compared colicin E1 alone. Combining these improvements in a mismatch repair proficient strain reduced counter-selection escape frequency by 1.3E6-fold in total, making tolC counter-selection as effective as most selectable markers, and adding a valuable tool to the genome editing toolbox. These improvements permitted us to perform stable and continuous rounds of selection/counter-selection using *tolC*, enabling replacement of 10 alleles without requiring genotypic screening for the first time. Finally, we combined these advances to create an optimized *E. coli* strain for genome engineering that is ∼10-fold more efficient at achieving allelic diversity than previous best practices.

## INTRODUCTION

Selectable markers have long been critical tools in molecular genetics, enabling the genetic manipulation of model organisms. Classical selectable markers are often antibiotic resistance genes, such as aminoglycoside phosphotransferase (kanamycin resistance), whose gene products are required for growth in media containing kanamycin. Selectable markers are used for plasmid maintenance, engineered conjugation and genome manipulations (1). In contrast, counter-selectable markers such as *sacB* (2) or barnase (3) are useful tools for different applications, such as plasmid curing (4,5), scar-less gene deletion (2) or engineering double-crossovers (6). However, counter-selectable markers often require stringent growth conditions to achieve robust counter-selection performance, and there are few means to ensure proper function of counter-selectable markers *in vivo*, which limit their application. Because selectable and counter-selectable markers have desirable and nonredundant uses, markers with both selectable and counter-selectable selection schemes (‘dual selectable markers’) are uniquely powerful and versatile. Dual selectable markers are particularly advantageous for generating gene replacements, scar-less genome editing and selection-coupled biosensors (7). Several dual selectable markers have been reported [*rpsL* (8,9), *galK* (10), *thyA* (11), *hsvTK* (12), *tetA* (13) and *tolC* (14)], but as with counter-selectable markers, dual-selectable markers suffer from high counter-selection escape and/or reliance on minimal media for robust counter-selection (10).

Without a suitable dual selectable marker, notable genome engineering projects have relied on cumbersome workarounds. For example, the Keio collection of *Escherichia coli* single-gene deletion clones was generated through *kanR* cassette replacement of each coding region, followed by FLP-based deletion of the *kanR* cassette (15), which scars the genome and risks off-target recombination elsewhere on the genome. In another example, separate selectable and counter-selectable markers were used together as a facsimile of a dual selectable maker to engineer protein substrate specificity and reactivity (3) without a means to ensure function of the counter-selectable marker. Finally, to minimize the *E. coli* genome, Posfai *et al.* (16) implemented a cloning-intensive method relying on I-*sceI*-induced double-strand break repair for scar-less serial deletion of genome segments. In each of these cases, a robust dual selectable marker would have been more convenient, suffered from less counter-selection escape, enabled scarless editing, and thus provided a more scalable approach for these or other more ambitious projects.

We were motivated to develop robust dual selectable markers to address these deficiencies, and to augment the power of Multiplex Automatable Genome Engineering (MAGE) (17). MAGE leverages λ Red recombineering in *E. coli* (18–20) to introduce mismatches, insertions and deletions onto the host genome, permitting exploration of evolutionary potential by rapidly generating combinatorial allelic diversity in a mixed population. Recent advances in MAGE have increased the average number of edits per MAGE cycle by reducing oligonucleotide degradation (1,21,22), manipulating the replisome (23) and co-selecting for recombinant genomes using Co-Selection MAGE (CoS-MAGE) (24,25). CoS-MAGE selects for recombinants by pairing a recombination to fix a broken selectable marker with nearby recombinations to introduce other edits of interest. Applying the associated selection leverages the significant linkage between nearby recombination events to enrich for highly modified clones after one cycle (24,25). However, MAGE is amenable to stable cycling, while CoS-MAGE using a selectable marker can only be performed once before the selectable marker must be inactivated anew. Without a robust dual selectable marker, CoS-MAGE is not amenable to repetitive cycling and requires time-intensive screening techniques that greatly limit its power and versatility.

We chose the dual selectable marker, *tolC,* as a test case for optimization because it is associated with convenient selections that can be performed in either liquid or solid, rich media (14). The *tolC* gene encodes a 1.5-kb monomer of a homotrimer pore (Supplementary Figure S1A). TolC is anchored in the outer membrane, spans the periplasm (PP) and provides a route for efflux of a wide variety of compounds. As summarized in Supplementary Figure S1B, TolC provides resistance to sodium dodecyl sulfate (SDS), and confers sensitivity to bactericidal colicin E1 (colE1) (26). While SDS selection is highly robust, *tolC*^+^ strains can readily escape from colE1-based counter-selection, for example, oligonucleotide-based deletion of *tolC* exhibits counter-selection escape rates of less than 0.01 up to 0.75, when tested at a variety of loci in the *E. coli* genome (14). These escape rates preclude the use of *tolC* for genomic manipulations that occur at frequencies lower than this range. To improve the counter-selection performance of *tolC*, we applied a generalizable, high-throughput workflow, including whole genome re-sequencing of 96 independent clones harboring a counter-selection escape phenotype and duplicating the genes whose loss-of-function alleles cause this phenotype. Additionally, we demonstrated that pairing vancomycin with colE1 reduces counter-selection escape frequency by requiring that escape mutations break both counter-selection mechanisms. Whereas continuous CoS-MAGE cycling using *tolC* was not possible owing to counter-selection escape, our improved strain and selection conditions allowed us to use *tolC* to rapidly converge on highly modified populations, offering one example of how a robust dual-selectable marker exhibiting minimal counter-selection escape will benefit many applications in molecular biology and genome engineering.

## MATERIALS AND METHODS

### Strains and culture methods

The strains used in this work were derived from EcNR2 (*Escherichia coli* MG1655 Δ*mutS::cat* Δ(*ybhB-bioAB*)::[λcI857 N(*cro-ea59*)::*tetR-bla*]) (17). We generated EcM1.0 (‘EcM’, *E. coli* MAGE-optimized) by inactivating the *xonA*, *exoX* and *xseA* nucleases (22) and by modulating primase activity [*dnaG*_Q576A, (23)]. We generated EcM2.0 by duplicating *tolQRA* at position 1 255 700 in EcM1.0. All strains were grown in liquid culture using the Lennox formulation of lysogeny broth (LB^L^) (27) with appropriate selective agents: carbenicillin (50 μg/ml), chloramphenicol (20 μg/ml), SDS (0.005% w/v) and vancomycin (64 μg/ml). During *tolC* counter-selections in liquid media, colicin E1 (colE1) was used at a 1:100 dilution from an in-house purification (28) that measured 14.4 μg_protein_/μl (1,29). Growth kinetics of representative *tolC^+^* and *tolC^−^* strains under these culture conditions are presented in the Supplementary Figures S1C–H).

### Colicin E1 agar plates

Clonal JC411 (28) isolates were picked, then passaged into 1 L LB^L^ production cultures. At OD_600_ = 0.1, we induced colicin E1 expression using 0.5 μg/ml of mitomycin-C, and then incubated cultures overnight at 37°C. Cultures were cooled on ice, then centrifuged at 9000 relative centrifugal force (rcf) for 10 min at 4°C. The pellets were resuspended in LB^L^, washed by centrifugation at 4000 rcf for 5 min at 4°C, and resuspended in 50 mM K_2_HPO_4_, pH 7.55. The pellets were sonicated on ice using a probe sonicator (Misonix Sonicator 3000), outputting 21–24 W, using 30 s on/30 s off cycles for 10 total minutes. Sonicates were clarified by centrifugation at 16 100 rcf for 5 min. These sonicates were added to molten LB^L^ agar + Carb (12.5 mL of sonicate per 1 L of media). The colicin plates were protected from light, stored at 4°C and exhibited a shelf life of ∼4 weeks.

### Oligonucleotides, polymerase chain reaction and isothermal assembly

A complete list of oligonucleotides used in this study is listed in Supplementary Table S6.

All polymerase chain reaction (PCR) products to be used in recombination or Sanger sequencing were amplified with Kapa Biosystems High-Fidelity polymerase, according to the manufacturer’s instructions. Multiplex allele-specific PCR (mascPCR) was used for multiplexed genotyping using the KAPA2G Fast Multiplex PCR Kit, according to previous methods (22,23). Sanger sequencing reactions were carried out through a third party (Genewiz, Eton Biosciences). To assemble multiple DNA fragments into a single contiguous sequence, we used isothermal assembly at 50°C for 60 min (30).

### Lambda red recombinations, MAGE and CoS-MAGE

Lambda red recombineering, the basis for MAGE and CoS-MAGE, was carried out as described previously (17,22,23). In singleplex recombinations, [oligo] = 1 μM. In <5-plex recombinations, [oligo]_each_ = 1 μM. In multiplexed recombinations (CoS-MAGE) with 10+ oligos, [selectable oligo] = 0.2 μM, whereas [nonselectable oligo]_total_ = 5 μM. Oligos were designed to hybridize to the lagging strand of the replication fork, as optimized previously (31). When double-stranded PCR products were recombined, 100 ng of double-stranded PCR product was used.

### CoS-MAGE modeling

**Figure 1.**
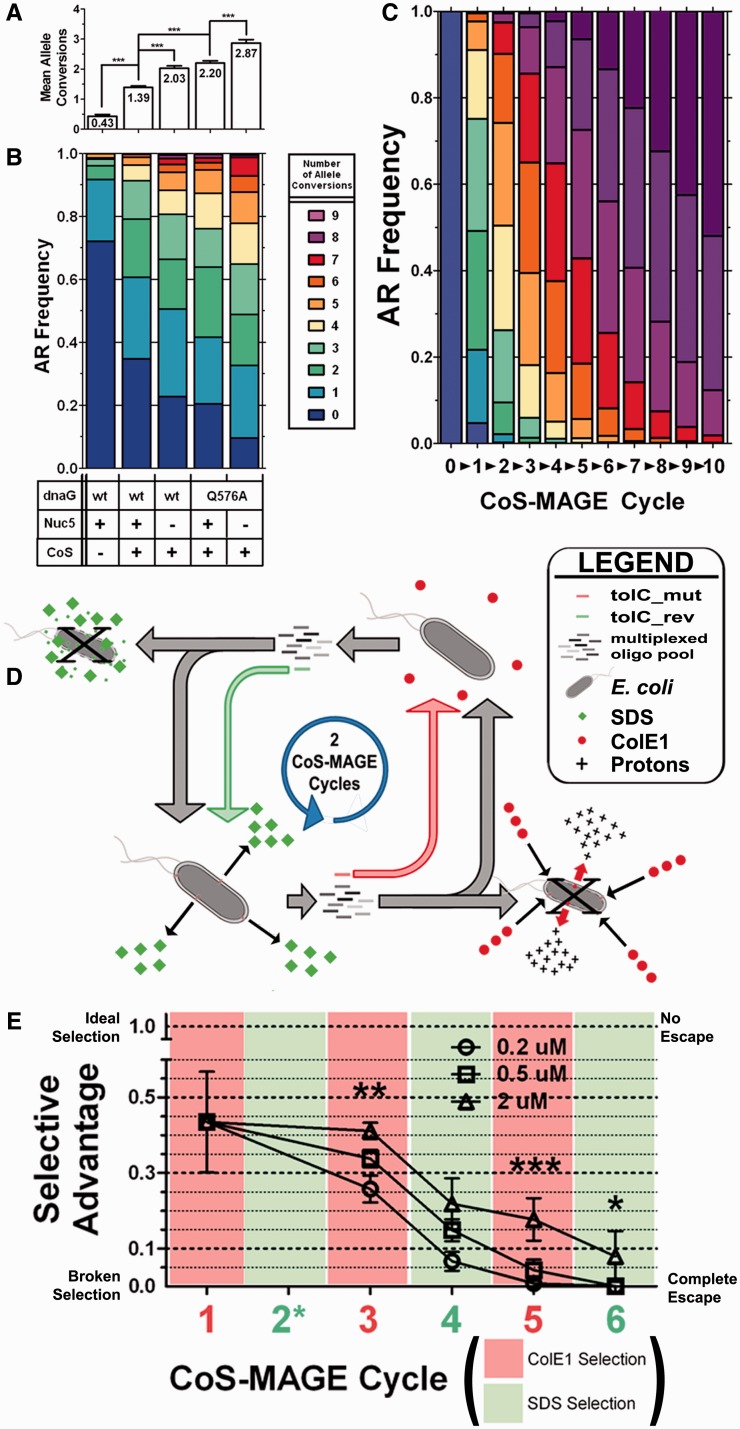
Repetitive *tolC* counter-selection rapidly generates a dysfunctional phenotype. (**A** and **B**). To motivate our work and demonstrate CoS-MAGE in improved strains, we averaged allele conversion data from recent studies (22,23) across 30 genomic loci [Sets 1–3 from (22,23)]. The data are reported as (A) Mean Allele Conversion ± SD of each population (*n* = 212, 821, 538, 561, 330, respectively) with the population mean reported within its respective bar, and (B) as a stacked bar graph where each color indicates the frequency of clones bearing that number of allele conversions. (**C**)**.** The data from strain EcNR2.Nuc5^-^.*dnaG*_Q576A were used to model the allele conversion distribution through 10 cycles of CoS-MAGE. This model did not account for any positional dependence for conversion of certain pairs of alleles (See Supplement and Figure S2). (**D**). A workflow diagram showing how the dual selectable marker, *tolC*, can be used for CoS-MAGE. Starting in the bottom left corner, the *tolC*^+^ genotype is recombined with a multiplexed oligo pool (gray oligos) plus the *tolC*_mut inactivation oligo (red). *tolC^−^* recombinants pass subsequent counter-selection in colE1, whereas the parental genotype is killed off (bottom right corner). Counter-selected *tolC^−^* population (top right corner) is then recombined with a multiplexed oligo pool (gray oligos) plus the *tolC*_rev reactivation oligo (green). *tolC*^+^ recombinants pass subsequent *tolC* selection (bottom left corner), whereas the parental *tolC^−^* genotype is killed (top left corner), and thus completes a tolC selection/counter-selection cycle (2 CoS-MAGE cycles). (**E**). Selection performance of CoS-MAGE cycling on EcNR2.2223749::*tolC* using three different concentrations of the selectable oligo (0.2, 0.5 and 2 µM) and the same concentration of the multiplexed, nonselectable oligos (5 µM), quantified as Normalize Selective Advantage (NSA, described in ‘Materials and Methods’ section) and presented as mean ± St. Dev. (*n* = 5+). Statistical significance was tested using a Kruskal–Wallis One-way ANOVA followed by Dunn’s test, where *P < 0.05, **P < 0.01 and ***P < 0.001. The plot background color indicates the selection (green) or counter-selection (red) step associated with that CoS-MAGE cycle. Over successive CoS-MAGE cycles, all three lineages escaped and NSA → 0.

The input data for CoS-MAGE modeling were the genotypes of the 10 targeted loci from 92 clones of a population of EcNR2.nuc5-.*dnaG*_Q576A cells that had been subjected to one cycle of CoS-MAGE (Figure 1AB, far right bar). The 92 genotypes from that data defined the probabilities of allele replacement (AR) patterns in a representative CoS-MAGE cycle. A complete description of the model is included in the Supplement.

### *tolC*-based selections

For recombinations inserting *tolC* or those reactivating *tolC*, cultures were recovered from electroporation for at least 1 h before applying the *tolC* selection using SDS. For recombinations deleting or inactivating *tolC*, cultures were recovered from electroporation for 5 hours, then passaged 1:100 into fresh LB^L^ for 2 hours before inoculating the counter-selection, using 1:100 colE1 (28). Growth kinetics of representative *tolC^+^* and *tolC^-^* strains under these culture conditions are presented in the (Supplementary Figure S1F and G). Kinetic monitoring of colE1 and SDS selections was performed on a shaking spectrophotometer (Spectramax M3, M5 or Biotek H4) at 34°C.

To quantify performance of liquid selections, we included a number of control selections that allowed us to devise a metric, called Normalized Selection Advantage (NSA, defined as 1 − [t^RS^ × (t^CNS^/t^RNS^)]/t^CS^), including recombinant cultures (‘R’) and control cultures (‘C’) into both selective (‘S’) and nonselective (‘NS’) media. Growth curves were analyzed for the minimum time, t, where OD_600_ ≥ 0.4. Thus, t^RS^/t^CS^ describes the growth advantage of recombinants in selective media (t^RS^), compared with negative controls in selective media (t^CS^). To normalize for disparate inoculums (due to variable culture growth or pipetting error), we divide by the corresponding ratio for nonselective media (t^RNS^/t^CNS^). When t^CS ^>> t^RS^, there is no growth of negative controls in the selection, 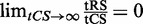, and NSA → 1. If the selection has failed and there is no selective advantage, then t^RS^ ∼ t^CS^, t^RS^/t^CS^ → 1, and NSA → 0.

### High-throughput sequencing of *tolC* counter-selection escape clones

To generate *tolC* counter-selection escape clones (SDS-resistant, colE1-resistant), we first cultured EcNR2.*tolC*^+^ in LB^L^ plus SDS. Confluent cultures were then passaged 1:100 into LB^L^ plus colE1 (counter-selection #1), then stamped into SDS (selection #2), then into colE1 (counter-selection #2), etc., until the fourth selection, after which each well was streaked onto SDS to isolate clones that were picked into LB^L^ plus SDS & colE1 for expansion and library preparation. Whole genome library preparation was carried out based on previously published protocols (32). Complete methods can be found in the Supplementary Methods. Sequencing was carried out on an Illumina HiSeq using 50 base pair paired-end reads, which yielded 6.46 × 10^7^ total reads. Raw reads were aligned to the *E. coli* K12 MG1655 reference genome (U00096) using BWA, and single nucleotide variants (SNVs) were called using the software tools GATK (33), SAMTools (34) and Freebayes (35) according to previously published methods (1). De-multiplexing the reads by barcode averaged 6.6 × 10^5 ^± 2.7 × 10^5^ reads/barcode (min, max: 1.95 × 10^5^, 1.56 × 10^6^), which translates into best-case read depth of 14.2 ± 5.8 (min, max: 4.2, 33.7). We identified 21 SNVs that deviated from reference in most of the 96 genomes (17 in 96 of 96, 2 in 95 of 96, 1 in 93 of 96 and 1 in 53 of 96 genomes), suggesting EcNR2-specific variants unrelated to the escape phenotype. These SNVs are reported in Supplementary Table S1.

## RESULTS AND DISCUSSION

### Motivations for CoS-MAGE cycling

Since 2009, MAGE has been the subject of constant technological development. We analyzed AR frequencies across 30 genomic loci on both replichores (1) to assess how AR frequencies have improved with recent strain modifications (Figure 1A). We chose to average AR frequencies for three 10-plex oligo pools to control for locus- and oligo-specific variance. The far left bar of Figure 1A shows the population distribution of edits in EcNR2 after a single cycle of MAGE. Importantly, 72% of the population is unmodified and only 8.3% of the population harbors more than a single edit, yielding a population average of 0.43 ± 0.06 edits/clone/cycle (Figure 1B), demonstrating that repeated cycling is crucial to attain complex genotypes with MAGE. Co-selection in EcNR2 halves the unmodified portion of the population (35%) and significantly increases the population average to 1.39 ± 0.05 edits/clone/cycle (***P < 0.0001, MAGE versus CoS-MAGE), confirming the significant linkage between selectable and nonselectable mutations (24). A single cycle of MAGE in EcNR2.nuc5-.*dnaG_Q576A* (22,23) resulted in 2.87 ± 0.11 mean edits/clone/cycle and only 9.6% of the population was unmodified (Figure 1A, right bar). Although MAGE performance in this strain is attractive, CoS-MAGE using selectable markers is not amenable to cycling and requires time-intensive screening between each CoS-MAGE cycle.

While MAGE has achieved highly modified genotypes through extensive cycling (1) and CoS-MAGE can achieve highly modified genotypes after a single cycle (23), thus we were interested to model continuous CoS-MAGE cycles using the AR frequency data gathered for one CoS-MAGE cycle using EcNR2.dnaG_Q576A (23). Our models (Figure 1C and Supplementary Figure S2) predict that >50% of the population would achieve a completely modified state (10 of 10 edits) after 10 cycles of CoS-MAGE, whereas MAGE in EcNR2 could only accomplish this after 90 cycles [Supplementary Figure S3 of (17)], suggesting that CoS-MAGE in optimized strains is roughly 10-fold more efficient than MAGE at achieving allelic diversity (please see Supplement for additional discussion of our models).

### Repetitive *tolC* counter-selection rapidly generates a dysfunctional phenotype

We reasoned that a dual selectable marker would enable a variety of convenient genome editing applications, including CoS-MAGE, but would require serially using both selection schemes in a continuous workflow. We envisioned that CoS-MAGE using the dual-selectable marker, *tolC*, would follow the workflow diagram in Figure 1D, which depicts a continuous cycle of inactivating tolC–performing the counter-selection (colE1)–restoring tolC–performing the selection (SDS)–etc. To test *tolC* as a dual selectable marker in CoS-MAGE cycling, we generated an EcNR2-based strain (naïve to the *tolC* counter-selection at the outset) as a test case. We began by deleting the endogenous *tolC* at nt 317 6137, then inserting a new *tolC* at nt 2 223 749 and using it to co-select for 10 TAG → TAA mutations [between 2 113 931 and 2 223 066 nt from (1)] in cycles of CoS-MAGE (Figure 1D). We quantified selection performance in these experiments by computing a Normalized Selective Advantage value (NSA, see ‘Materials and Methods’ section for discussion of the metric and its features/drawbacks). NSA = 1 signifies perfect selection with no negative control escape (Supplementary Figure S3A, left panel), and NSA = 0 indicates complete selection escape (Supplementary Figure S3A, right panel).

We conducted five replicates of counter-selection-coupled, endogenous *tolC* deletion using 5 × 10^5^ cells and observed substantial escape (NSA = 0.44 ± 0.13, Cycle 1, Figure 1E) after a single counter-selection. We inserted the new *tolC* at nt 2 223 749 and continued with CoS-MAGE cycles using different concentrations of selectable oligos (0.2, 0.5 and 2 µM), which exhibited statistically significantly different NSAs (**P < 0.01, 2 μM versus both 0.5 and 0.2 μM). Higher concentrations of selectable oligo correlated with higher NSA, consistent with an increased mole fraction of an oligo increasing its allele recombination (AR) frequency within a multiplexed pool (24). Over subsequent cycles, NSA continued to decrease and the 0.2 and 0.5 μM lineages completely escaped at Cycle 5 (0.01 ± 0.02 and 0.03 ± 0.06, respectively; *P* = n.s., 0.2 versus 0.5 μM), whereas the 2 μM lineage completely escaped at Cycle 6 (0.06 ± 0.06; *P < 0.05, 2 μM versus 0.5 and 0.2 μM). These data suggest that *tolC* could not be used for repetitive selection/counter-selection schemes.

Supplemental experiments (Supplementary Figure S3) supported the hypothesis that counter-selection escape was mutational and not due to colE1 degradation or loss of activity (Supplementary Figure S3B). Sanger sequencing confirmed that the *tolC* coding region was intact. Thus, we hypothesized that whole-genome re-sequencing could shed light on counter-selection escape strategies.

### High-throughput sequencing diagnosis of *tolC* counter-selection escape

**Figure 2.**
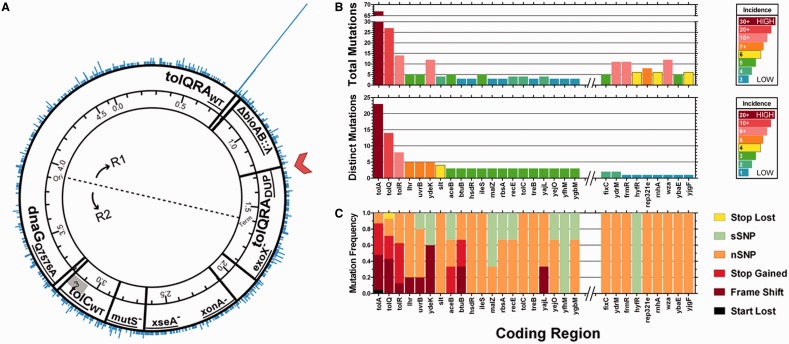
High-throughput sequencing diagnosis of *tolC* counter-selection escape. (**A**). SNVs were binned into 2 kb bins along the genome from 0 to 4.6 Mb. These data are presented on the outermost ring in a circular bar format, where the position of a bar is indicative of the bin in which a mutation falls and the y-axis is mutations/bin. Other notable genomic features of these strains are marked in the inner ring, including duplication of the *tolQRA* operon (incl. *ybgC*), at position 1 255 700 (red chevron), the endogenous *tolQRA* operon (*tolQRA_WT_*), λ prophage insertion at the *bioAB* locus (Δ*bioAB*::λ), inactivated nucleases (*exoX^−^, xonA^−^, xseA^−^*), *mutS* inactivation (*mutS^−^*), endogenous *tolC (*tolC_WT_) and the primase mutation (*dnaG_Q576A_*). A kb-scale version of this figure is presented in Supplementary Figure S3D, which gives resolution on the mutations that fall around the *tolQRA* operon (chr: 763 373–786 830). (**B**). SNVs were categorized by coding region (x-axis) and plotted versus Total Mutations (top panel), and by Distinct Mutations (bottom panel). Total Mutations was defined as the Distinct Mutations times incidence. The x-axes in both panels are sorted by the number of Distinct Mutations (bottom panel) from most [*tolA*, (23)] to least [*frmR, hyfR, rep321e, rnhA, wza, ybaE, yjgF*, (1)]. The x-axes are broken for the purposes of presentation, excluding coding regions exhibiting 1 or 2 Distinct Mutations. Among the coding regions exhibiting 1 or 2 Distinct Mutations, *fixC, ydrM, frmR, hyfR, rep321e, rnhA, wza, ybaE, yjgF* were the only coding regions exhibiting 5+ Total mutations (e.g. detected in multiple independent genomes) and are presented to the right of the break in the x-axis. At the right of both panels, mutation incidence is coded as a panel-specific heat map (blue for low incidence, red for high incidence) with panel-specific scaling indicated therein as the threshold value for inclusion. (**C**) Frequency of Mutation Type was calculated based on variant calls from SNPEff (33), including Start Lost (black), Frame Shift (dark red), Stop Gained (red), nSNV (nonsynonymous SNV, orange), sSNV (synonymous SNV, light green) and Stop Lost (yellow) and plotted on the same broken x-axis as in (B).

**Figure 3.**
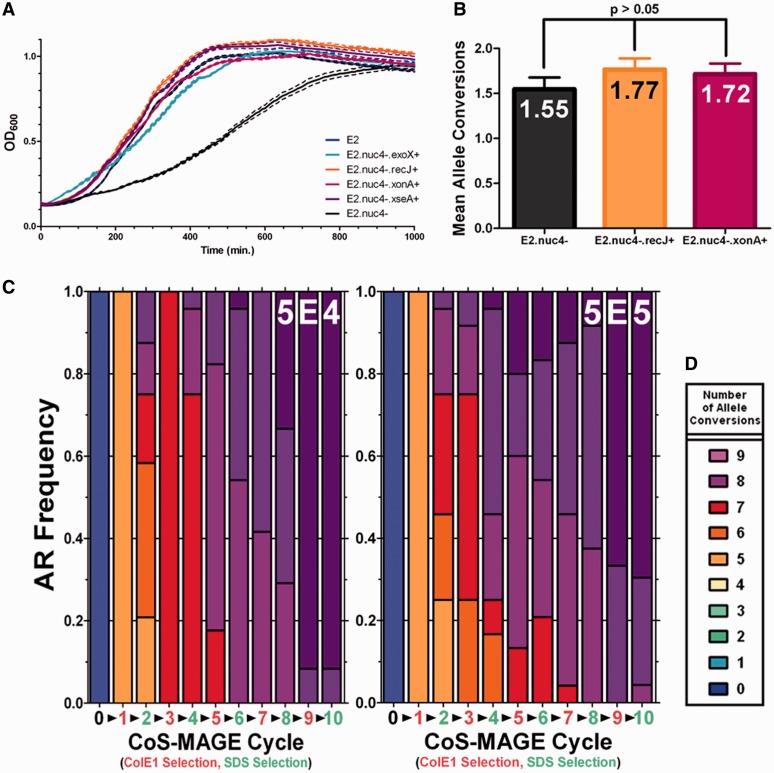
*tolQRA* duplication enables stable CoS-MAGE cycling. (**A**). To probe the post-recombination growth phenotype of Nuc5^−^-based strains, we individually reverted each of the four inactivated nucleases: *exoX*^+^ (cyan); *recJ*^+^ (orange); *xonA*^+^ (red); *xseA*^+^ (purple). As controls, we included EcNR2 (Nuc5^+^, blue) and EcNR2.Nuc5*^−^* (black). To study the poor post-recombination recovery phenotype associated with EcNR2.Nuc5^−^, we recombined these six strains with a 5.2 µM multiplexed oligo pool, then monitored growth post-recombination. (**B**). To understand whether nuclease reversion results in inferior CoS-MAGE performance to Nuc5^−^, we tested Nuc5^−^, the *recJ* reversion (*recJ*^+^) and the *xonA* reversion (*xonA*^+^) strains in a single cycle of CoS-MAGE. The mascPCR data are presented as Mean Allele Conversion ± SD. Statistical analysis (Kruskal–Wallis ANOVA) revealed that the means were not statistically significantly different (*P* > 0.05). Moving forward, we implemented the *recJ* reversion in EcM2.0 (EcNR2.*dnaG*_Q576A.*xseA*-.*exoX*-.*xonA*-.1255700::*tolQRA*). (**C** and **D**). EcM2.0 was subjected to continuous CoS-MAGE cycling of Oligo Set 1 (22,23) using the endogenous *tolC_WT_*. We inoculated selections (SDS) using 5 × 10^6^ cells/well, and counter-selections using 5 × 10^4^ cells/well (C) or 5 × 10^5^ cells/well (D). After each respective selection, clones were plated and screened for allele conversions at the 10 loci of interest using mascPCR.

To identify alleles that cause the *tolC* counter-selection escape, we re-sequenced the genomes of 96 independent *tolC* counter-selection escape clones (see ‘Materials and Methods’ section). Our analysis relies on re-sequencing many independent counter-selection escape clones to categorize genomic variants into those that are causal and those that are unrelated to counter-selection escape. Across the 96 genomes, there was an enrichment (108 of 3272 total mutations in the data set) for mutations in a 4-kb window from 774 000 to 778 000 nt, corresponding to the *tolQRA* operon (Figure 2A and Supplementary Figure S3D). *tolQ* contained 14, *tolR* contained 8, and *tolA* contained 23 distinct mutations in their respective coding regions (Figure 2B, bottom panel), yet zero mutations were identified in *ybgC*, also encoded by the *tolQRA* operon. After *tolQ/tolR/tolA*, *lhr, ydeK* and *uvrB* contained the next most distinct mutations each (*n* = 5), consistent with their large coding sizes (4687*,* 3978 and 2022 bp, respectively). The *tolQ*, *tolR* and *tolA* mutations were enriched for start codon mutations, premature stop codons and frameshifts (87% of all distinct mutations in *tolA*, 71% for *tolQ* and 63% for *tolR*, Figure 3C). Aside from the *tolQRA* operon, no other 4 kb window contained >13 total mutations, but there were a number of distinct mutations that were observed in multiple independent genomes (Figure 2B, columns to right of break in x-axis), necessitating validation. Finally and perhaps most indicative of causality, 89 of 96 genomes in the dysfunctional clone set contained at least one mutation in *tolQRA*.

Of the seven remaining dysfunctional genomes that lacked *tolQRA* mutations, two genomes shared the same nonsynonymous *tolC* L235P mutation, which is located on the exterior face of the PP-spanning equatorial domain of *tolC*, which protrudes from the exterior of the channel. Based on docking models (36), this protrusion is involved in stabilizing protein–protein interactions between TolC and its active transport systems in the IM (e.g. *acrAB*). We posit that the kink in the polypeptide backbone contributed by proline may interfere with interactions between TolC and TolA, perhaps making TolA less available for colE1 binding (37). The five remaining genomes did not share any common mutations, suggesting rare escape mechanisms.

### Assessing causality of alleles identified via high-throughput sequencing

To functionally assess the alleles identified by sequencing, we designed MAGE oligos to generate the 55 most abundant mutations (XM oligos), and to knockout the 20 most frequently mutated coding regions (cvrALL oligos) seen in our data set. Each of these oligos was recombined into EcNR2.*tolC*^+^ and counter-selected using colE1. We hypothesized that oligos that confer mutations causing *tolC* counter-selection escape will shorten the culture time in colE1 that is required to reach OD_600_ = 0.4, as a larger portion of the preselection population would be resistant to colE1. Presented in Supplementary Table S2, we quantified relative causality by including multiple controls, both background (mock recombination) and positive (tolC knockout recombination), which were used to calculate Normalized Culture Time (see Supplemental Methods for Definition of this metric). An oligo exhibiting a Normalized Culture Time of ∼0 encodes an escape mutation, whereas an oligo exhibiting a Normalized Culture Time of ∼1 encodes an unrelated mutation. We acknowledge that the oligo-dependent variance in AR frequency seen in MAGE (1,17) will introduce an additional variable to our readout, leading to increased Type II errors.

Normalized Culture Time for all putative dysfunctional oligos fell between −0.13 ± 0.01 and 1.31 ± 0.12 (Supplementary Table S2). As expected, the cvrALL_*tolC*_3 oligo generates colE1 resistance through *tolC* knockout with a Normalized Culture Time of 0.01 ± 0.01. Causal mutations (defined as Normalized Culture Time < 0.6) included four frameshifts in *tolA*, 1 SNV in *tolA*, 2 frameshifts in *tolR,* 2 SNVs in *tolR*, as well as the cvrALL oligos for *tolQ* and *tolR*. The cvrALL oligo for *tolA* led to a Normalized Culture Time of only 0.73 ± 0.11, possibly because recombination frequency is low for this oligo or because in-frame ATG codons (such as M54, M67 or M83) rescue *tolA* translation downstream of the premature stop introduced by the oligo. Borderline mutations (defined as 0.6 ≤ Normalized Culture Time < 1.0) included two frameshifts in *tolA*, one frameshift in *tolQ* and the cvrALL oligo for *tolA*. Examples where we invalidated borderline mutations like *ogt*, *recR* and *treB* can be found in the Supplement. The remainder of the 75 tested MAGE oligos produced Normalized Culture Times ≥ 1, suggesting that they are not involved in counter-selection escape.

The domain structure of the 421 amino acid TolA protein corresponds to a single pass transmembrane protein with a tight, α-helical domain (38) extending into the PP. Published work has shown that a −1 frameshift in *tolA* after I400 led to colE1 resistance (37), which agrees well with our data set showing a prevalence of *tolA* frameshifts that led to colE1 resistance (including XM_*tolA*_776642_GGCAA_G where translation falls out of frame after G359; Supplementary Table S2), and suggests that colE1 engages the C-terminus of *tolA*. This mechanistic insight is further supported by the fact that 67 out of a total of 108 *tolQRA* mutations (62.0%) were in *tolA* (Figure 2B, top panel), which is a slight enrichment over the frequency expected based on coding region size alone (53.0%). Beyond the colE1-TolA interaction, proper activity of the entire TolQRA complex is required, as suggested previously (39) and as evinced by the causal cvrALL mutations for *tolQ* and *tolR* (Supplementary Table S2). TolQ has been implicated as a molecular motor for the Tol complex (39,40), while TolR is required to stabilize TolQ in the IM (39). In fact, TolR_D23_ mutations, which were previously shown to abolish the TolQ-TolR interaction and increase TolQ turnover [TolR_D23A_ and TolR_D23R_ (39)], were also seen in this data set (TolR_D23G_) and were determined to be causal when introduced by XM_*tolR*_775139_A_G (Supplementary Table S2). Taken together, these results validate the hypothesis that loss-of-function mutations in *tolQRA* lead to *tolC* counter-selection escape.

### Engineering a strain with improved *tolC* counter-selection

Based on a mechanistic understanding of *tolC* counter-selection escape born from whole-genome re-sequencing, we hypothesized that duplicating the *tolQRA* operon would safeguard against loss-of-function mutations and make the *tolC* counter-selection more robust (Figure 3C). To guide insertion of the duplicated *tolQRA* operon, candidate destinations were chosen to be separated from the wild-type operon by at least one essential gene, reducing the chances of *recA*-mediated recombination between identical operons. We generated the *tolQRA*-duplicated strain, EcNR2.1255700::*tolQRA*.*tolC^+^* and inoculated 32 replicates of 10^6^ mid-log EcNR2.*tolC^+^* or EcNR2.1255700::*tolQRA*.*tolC^+^* (*tolQRA* duplicated) cell into LB^L^ plus colE1 to analyze growth. All EcNR2.tolC^+^ replicates escaped, attaining OD_600_ = 0.4 at 781.1 ± 18.2 min (mean ± stdev), whereas only 1 of the 32 EcNR2.1255700::*tolQRA* replicates grew over the 48 h course (OD_600_ = 0.4 at 919.5 min). This demonstrates that *tolQRA* duplication protects against *tolC* counter-selection escape. Importantly, *tolQRA* duplication led to no apparent phenotypes in growth or recombination.

### *tolQRA* duplication enables stable CoS-MAGE cycling

Leveraging reduced *tolC* counter-selection escape, we attempted to perform continuous stable CoS-MAGE cycling on a *tolQRA*-duplicated version of a recently optimized strain (22,23). However, because Nuc5^−^-based strains exhibit a slow recovery phenotype after recombination (22), this modification is not suited for cycling. By individually reverting each inactivated nuclease in Nuc5^−^, we determined that *recJ* reversion reduced this recovery phenotype (Figure 3A) without compromising the improved MAGE performance seen in the Nuc5^-^ background (Figure 3B, see Supplement for our complete line of reasoning and discussion of these data). We defined EcNR2.Δ*tolC*.*dnaG*_Q576A.*exoX*^-^.*xonA*^-^.*xseA*^-^ as EcM1.0 (for *E. coli* MAGE-optimized), then duplicated *tolQRA* in EcM1.0 to produce EcM2.0.

To test CoS-MAGE cycling in EcM2.0, we cycled the endogenous *tolC* to co-select for 10 nearby oligo-encoded TAG to TAA mutations [Set #1 from (22)]. We performed *tolC* selections (SDS) using 5 × 10^6^ cells/selection, and performed *tolC* counter-selection (colE1) using 5 × 10^4^ (Figure 3C), 5 × 10^5^ (Figure 3D), 5 × 10^6^ cells/counter-selection to test if all three counter-selection inocula would support continuous and stable cycles of selection/counter-selection. The first two lineages maintained ideal selections for 10+ cycles, whereas the 5 × 10^6^ cells/counter-selection lineage escaped during the third counter-selection. MascPCR data from the two lineages exhibiting ideal selections (Figure 3CD) showed that both rapidly moved through the recoding landscape from unmodified (at Cycle 0) to completely modified (at Cycle 10), with 92% of the 5 × 10^4^ lineage and 70% of the 5 × 10^5 ^lineage exhibiting 10 of 10 conversions. There was more diversity in the 5 × 10^5^ lineage, consistent with larger counter-selection library size, while the 5 × 10^4^ lineage often collapsed diversity to a single genotype (compare odd cycle #’s of Figure 3C and D, please see the Supplement for additional discussion of these results in the context of our model). Taken together, these data suggest that the *tolC* counter-selection in *tolQRA*-duplicated lineages such as EcM2.0 supports stable CoS-MAGE cycling, but that a careful balance between library complexity and escape frequency must be maintained.

### Other approaches to reduce tolC counter-selection escape

**Figure 4.**
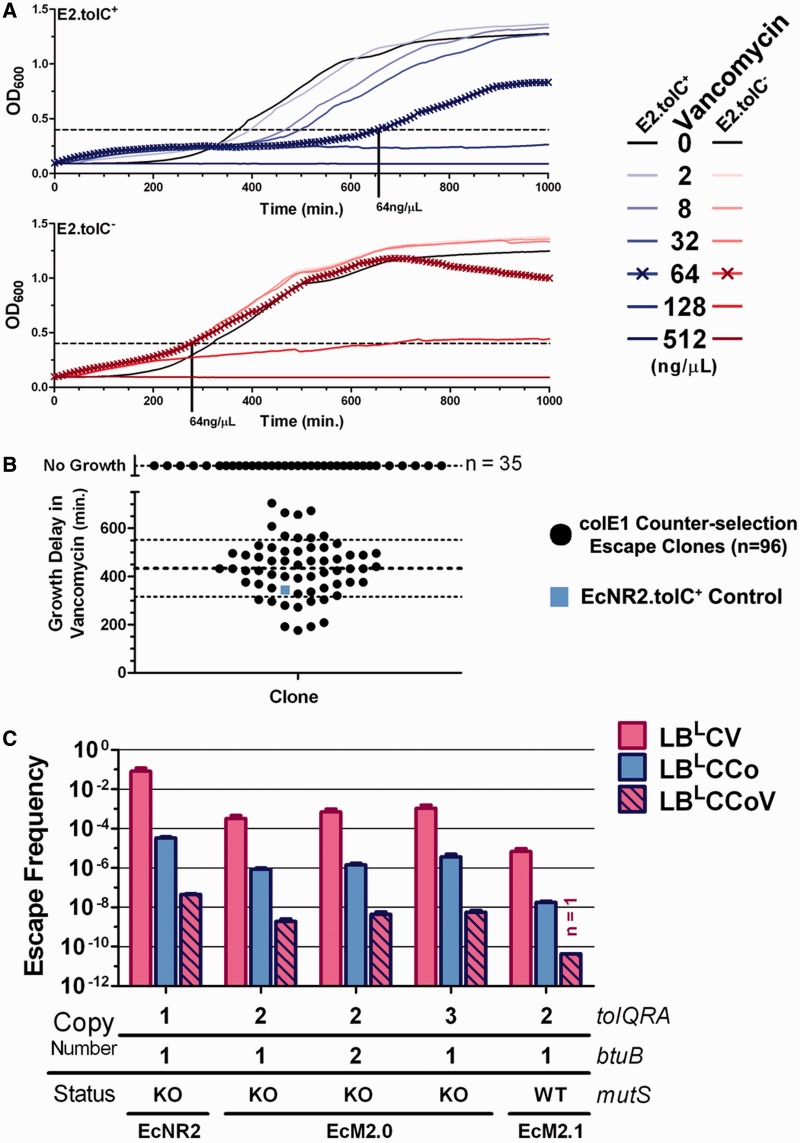
Other approaches to reduce tolC counter-selection escape. (**A**) We tested the *tolC*-dependence of vancomycin sensitivity on EcNR2.*tolC*^+^ (top panel, blue) and EcNR2.*tolC*^−^ (bottom panel, red) by kinetically measuring growth across a 2-fold dilution series from 2 µg_vanc_/µl (lightest curve) to 512 µg_vanc_/µl (darkest curve). At 64 µg_vanc_/µl (curves marked with ‘x’), EcNR2.*tolC*^−^ cells grew normally, whereas EcNR2.*tolC*^+^ growth was impaired, leading to a maximal growth advantage. (**B**) To test mechanistic independence of counter-selection in vancomycin from that of colE1, we cultured the escape clones used for whole genome re-sequencing (*n* = 96, 10^6^ cells/well, black circles) with or without 64 µg_vanc_/µl. The data are presented as the Growth Delay (in minutes) in vancomycin with respect to no vancomycin. Many clones (*n* = 35) did not show any growth within the 48-h kinetic experiment and were plotted as ‘No Growth’ above the broken y-axis. Of the clones that did grow (*n* = 61), the mean (heavy dashed line) and standard deviation (light dashed line) of Growth Delay are plotted with the data. An EcNR2.*tolC*^+^ control was also tested (blue square), to show how vancomycin delays naïve *tolC*^+^ cells. (**C**). To quantify our improvements to the *tolC* counter-selection, we measured escape frequency by plating known amounts of *tolC^+^* strains onto vancomycin plates (LB^L^CV), colicin E1 plates (LB^L^CCo) and colicin E1/vancomycin plates (LB^L^CCoV). Data were gathered by counting colonies from at least four independent biological replicates and are presented as Mean ± SEM, except for EcM2.1 on LB^L^CCoV, which is based on a single data point (1 escape clone out of 2.32 × 10^10^ total cells plated).

Since *tolC* has been implicated in efflux of a variety of compounds (electrolytes, ions, antibiotics, detergents, etc.), we searched for other *tolC* counter-selection agents that use a different mechanism than colE1. Assuming mechanistic independence, the escape frequency of the simultaneous application of two orthogonal counter-selection agents would be the product of the escape frequencies of each individual agent, thereby increasing the stringency of counter-selection. Vancomycin is an amino-glycoside antibiotic that inhibits the D-Ala-D-Ala Ligase and requires *tolC* to gain access to the PP in gram-negative bacteria (41). We tested the *tolC*-dependence of vancomycin across a dilution series from 0.5 to 512 µg/ml, using 10^6^ EcNR2 cells (Figure 4A, top panel) or 10^6^ EcNR2.Δ*tolC* cells (Figure 4A, bottom panel). EcNR2.Δ*tolC* exhibited an optimal selective advantage ∼7 doublings (377 minutes) in 64 µg_vancomycin_/ml. We demonstrated the mechanistic independence of vancomycin escape from colE1 escape by showing that vancomycin selection was still effective on the 96 *tolC* counter-selection escape clones used for whole-genome re-sequencing (Figure 4B). Thirty-five clones exhibited no growth over 24 h, and the 61 clones that grew averaged a delay of 433 ± 118 min (minimum 176 min) with respect to nonselective media controls, similar to that of a colE1-naïve EcNR2 control (Figure 4B, blue square). These results suggest that applying colE1 and vancomycin together could further reduce *tolC* counter-selection escape.

Other approaches to improve the *tolC* counter-selection included increasing TolC turnover and restoring *mutS* mismatch repair. We attempted to ssrA tag *tolC* to reduce recovery time before *tolC* counter-selection, but found it difficult to balance adequate expression levels with quick turnover (Supplementary Figure S5, see Supplement for further discussion of these experiments). Mismatch repair deficiency improves λ Red-mediated AR frequencies (21), but also increases mutagenesis by 10- to 100-fold (42), thereby increasing the frequency at which *tolC* counter-selection escape mutations arise. For facile restoration of mismatch repair as needed, we restored *mutS* in place of Δ*mutS::cat* by selecting for insertion of *tolC* coupled to the 1.2 kb N-terminus of *mutS*, then counter-selecting for replacement of *tolC* using the 1.5 kb C-terminus of *mutS,* and finally using MAGE to inactivate the restored coding region to re-enable MAGE. This strain is designated EcM2.1.

After implementing all strain improvements, we quantified *tolC* counter-selection escape frequencies with agar plates containing colE1 (Figure 4C), prepared as described in the ‘Materials and Methods’ section, and validated as described in the (Supplementary Figure S6). EcNR2 escaped counter-selection at a frequency of 3.4E-5 ± 5E-6 on colE1 agar plates (LB^L^CCo), while *tolQRA* duplicated lineages (EcM2.0) exhibited a 40-fold reduction in escape frequency (8.5E-7 ± 1.4E-7). Notably, triplicated *tolQRA* or duplicated *btuB* strains did not exhibit reduced escape frequencies compared with EcM2.0, suggesting higher copy number does not offer additional protection from *tolC* counter-selection escape. While the escape frequencies for agar plates containing only vancomycin (LB^L^CV) were very high, for example 8.3E-2 ± 3.6E-2 in EcNR2, escape frequencies for plates containing colicin and vancomycin (LB^L^CCoV) were very low, for example 2.0E-9 ± 6E-10 in EcM2.0, which was a ∼400-fold reduction in escape frequency from that on LB^L^CCo and a 170 000-fold reduction from LB^L^CV alone. Platings on LB^L^CV also suggested that *tolQRA* duplication (EcM2.0-based strains) is associated with a ∼150-fold reduction in vancomycin escape, despite seemingly independent mechanisms (Figure 4B). Finally, *mutS* reactivation in EcM2.1 led to the lowest observed escape frequency (4.3E-11, or 1 clone in 2.3E10 total cells plated, Figure 4C), making *tolC* counter-selection as effective as many selectable markers. Low escape frequencies will support larger library sizes in the *tolC* counter-selection and enable the use of *tolC* for inefficient genomic manipulations, such as conjugal transfer of large genome segments (1).

## CONCLUSIONS

Robust selectable markers are essential for basic molecular biology research as well as genome engineering applications (1,25,29). To support these efforts, we have developed an extensible workflow to diagnose mechanisms of selection escape and have developed several strategies to improve selection robustness. Based on our experience with dual selectable markers such as *galK* (10), *thyA* (11), *hsvTK* (12) and *tetA* (13), we chose *tolC* (14) as a test case because of convenient selection (SDS) and counter-selection (colE1) schemes. We used whole-genome re-sequencing for the unbiased identification of genes involved in *tolC* counter-selection escape. The results were consistent with biochemical studies (37–39) indicating that *tolQ*, *tolR* and *tolA* are involved in colE1 import, but surprisingly indicated no likely role for *btuB* (40). Based on these results, we found that *tolQRA* duplication, but not *btuB* duplication, reduced *tolC* counter-selection escape frequency by 40-fold. We further reduced *tolC* counter-selection escape ∼425-fold by using vancomycin together with colE1. In EcM2.1 (*mutS*^+^), this resulted to a ∼1E-11 counter-selection escape frequency, which totals a 1.3E6-fold improvement over our initial methods. Similar to how vancomycin and colE1 synergize to improve the *tolC* counter-selection, our colleagues (43) recently published on a dual-selectable, *tetA-sacB* tandem cassette where fusaric acid and sucrose synergize to achieve more robust counter-selection than either marker alone. Robust dual-selectable markers, like *tolC* and *tetA-sacB,* are welcome and complementary tools for genome editing. It will be interesting to compare these systems (and others as they emerge), as fundamental aspects of each system will dictate relative performance. To conclude, we have created an optimized strain for genome engineering, called EcM2.1 (*E. coli* MAGE-optimized 2.1), on which we performed stable, continuous cycles of CoS-MAGE using *tolC* to generate a completely modified population of cells possessing 10 of 10 desired modifications without requiring any intermittent screening or direct selection. This unprecedented capability will facilitate repetitive selection/counter-selection cycles using large library sizes, which will be an important tool for genome editing and synthetic biology.

## ACCESSION NUMBERS

We will deposit our Illumina sequencing data set into the NCBI Short Read Archive.

## SUPPLEMENTARY DATA

Supplementary Data are available at NAR Online.

## FUNDING

US Department of Energy [DE-FG02-02ER63445]; US Defense Advanced Research Projects Agency [N66001-12-C-4211 to F.J.I.]; US Department of Defense NDSEG Fellowship (to M.J.L); US National Science Foundation Graduate Research Fellowship (to D.B.G.); and Arnold & Mabel Beckman Foundation (to F.J.I.). Funding for open access charge: US Department of Energy [DE-FG02-02ER63445].

*Conflict of interest statement*. None declared.

## Supplementary Material

Supplementary Data

## References

[1] Isaacs FJ, Carr PA, Wang HH, Lajoie MJ, Sterling B, Kraal L, Tolonen AC, Gianoulis TA, Goodman DB, Reppas NB (2011). Precise manipulation of chromosomes *in vivo* enables genome-wide codon replacement. Science.

[2] Ried JL, Collmer A (1987). An nptI-sacB-sacR cartridge for constructing directed, unmarked mutations in gram-negative bacteria by marker exchange-eviction mutagenesis. Gene.

[3] Wang L, Schultz PG (2001). A general approach for the generation of orthogonal tRNAs. Chem. Biol..

[4] Hynes MF, Quandt J, O'Connell MP, Puhler A (1989). Direct selection for curing and deletion of Rhizobium plasmids using transposons carrying the *Bacillus subtilis* sacB gene. Gene.

[5] Stojiljkovic I, Trgovcevic Z, Salaj-Smic E (1991). Tn5-rpsL: a new derivative of transposon Tn5 useful in plasmid curing. Gene.

[6] Marx CJ (2008). Development of a broad-host-range sacB-based vector for unmarked allelic exchange. BMC Res. Notes.

[7] Dietrich JA, McKee AE, Keasling JD (2010). High-throughput metabolic engineering: advances in small-molecule screening and selection. Ann. Rev. Biochem..

[8] Stavropoulos TA, Strathdee CA (2001). Synergy between tetA and rpsL provides high-stringency positive and negative selection in bacterial artificial chromosome vectors. Genomics.

[9] Bird AW, Erler A, Fu J, Heriche JK, Maresca M, Zhang Y, Hyman AA, Stewart AF (2012). High-efficiency counterselection recombineering for site-directed mutagenesis in bacterial artificial chromosomes. Nat. Methods.

[10] Warming S, Costantino N, Court DL, Jenkins NA, Copeland NG (2005). Simple and highly efficient BAC recombineering using galK selection. Nucleic Acids Res..

[11] Wong QN, Ng VC, Lin MC, Kung HF, Chan D, Huang JD (2005). Efficient and seamless DNA recombineering using a thymidylate synthase A selection system in *Escherichia coli*. Nucleic Acids Res..

[12] Tashiro Y, Fukutomi H, Terakubo K, Saito K, Umeno D (2011). A nucleoside kinase as a dual selector for genetic switches and circuits. Nucleic Acids Res..

[13] Stavropoulos TA, Strathdee CA (2000). Expression of the tetA(C) tetracycline efflux pump in *Escherichia coli* confers osmotic sensitivity. FEMS Microbiol. Lett..

[14] DeVito JA (2008). Recombineering with tolC as a selectable/counter-selectable marker: remodeling the rRNA operons of *Escherichia coli*. Nucleic Acids Res..

[15] Baba T, Ara T, Hasegawa M, Takai Y, Okumura Y, Baba M, Datsenko KA, Tomita M, Wanner BL, Mori H (2006). Construction of *Escherichia coli* K-12 in-frame, single-gene knockout mutants: the Keio collection. Mol. Syst. Biol..

[16] Posfai G, Plunkett G, Feher T, Frisch D, Keil GM, Umenhoffer K, Kolisnychenko V, Stahl B, Sharma SS, de Arruda M (2006). Emergent properties of reduced-genome *Escherichia coli*. Science.

[17] Wang HH, Isaacs FJ, Carr PA, Sun ZZ, Xu G, Forest CR, Church GM (2009). Programming cells by multiplex genome engineering and accelerated evolution. Nature.

[18] Zhang Y, Buchholz F, Muyrers JP, Stewart AF (1998). A new logic for DNA engineering using recombination in *Escherichia coli*. Nat. Genet..

[19] Yu D, Ellis HM, Lee EC, Jenkins NA, Copeland NG, Court DL (2000). An efficient recombination system for chromosome engineering in *Escherichia coli*. Proc. Natl Acad. Sci. USA.

[20] Datsenko KA, Wanner BL (2000). One-step inactivation of chromosomal genes in Escherichia coli K-12 using PCR products. Proc. Natl Acad. Sci. USA.

[21] Wang HH, Xu G, Vonner AJ, Church G (2011). Modified bases enable high-efficiency oligonucleotide-mediated allelic replacement via mismatch repair evasion. Nucleic Acids Res..

[22] Mosberg JA, Gregg CJ, Lajoie MJ, Wang HH, Church GM (2012). Improving lambda red genome engineering in *Escherichia coli* via rational removal of endogenous nucleases. PLoS One.

[23] Lajoie MJ, Gregg CJ, Mosberg JA, Washington GC, Church GM (2012). Manipulating replisome dynamics to enhance lambda Red-mediated multiplex genome engineering. Nucleic Acids Res..

[24] Carr PA, Wang HH, Sterling B, Isaacs FJ, Lajoie MJ, Xu G, Church GM, Jacobson JM (2012). Enhanced multiplex genome engineering through co-operative oligonucleotide co-selection. Nucleic Acids Res..

[25] Wang HH, Kim H, Cong L, Jeong J, Bang D, Church GM (2012). Genome-scale promoter engineering by coselection MAGE. Nat. Methods.

[26] Jakes KS, Cramer WA (2012). Border crossings: colicins and transporters. Ann. Rev. Genet..

[27] Lennox ES (1955). Transduction of linked genetic characters of the host by bacteriophage P1. Virology.

[28] Schwartz SA, Helinski DR (1971). Purification and characterization of colicin E1. J. Biol. Chem..

[29] Lajoie MJ, Rovner AJ, Goodman DB, Aerni HR, Haimovich AD, Kuznetsov G, Mercer JA, Wang HH, Carr PA, Mosberg JA (2013). Genomically recoded organisms expand biological functions. Science.

[30] Gibson DG, Young L, Chuang RY, Venter JC, Hutchison CA, Smith HO (2009). Enzymatic assembly of DNA molecules up to several hundred kilobases. Nat. Methods.

[31] Ellis HM, Yu D, DiTizio T, Court DL (2001). High efficiency mutagenesis, repair, and engineering of chromosomal DNA using single-stranded oligonucleotides. Proc. Natl Acad. Sci. USA.

[32] Rohland N, Reich D (2012). Cost-effective, high-throughput DNA sequencing libraries for multiplexed target capture. Genome Res..

[33] DePristo MA, Banks E, Poplin R, Garimella KV, Maguire JR, Hartl C, Philippakis AA, del Angel G, Rivas MA, Hanna M (2011). A framework for variation discovery and genotyping using next-generation DNA sequencing data. Nat. Genet..

[34] Li H, Handsaker B, Wysoker A, Fennell T, Ruan J, Homer N, Marth G, Abecasis G, Durbin R (2009). The Sequence Alignment/Map format and SAMtools. Bioinformatics.

[35] Barnett DW, Garrison EK, Quinlan AR, Stromberg MP, Marth GT (2011). BamTools: a C++ API and toolkit for analyzing and managing BAM files. Bioinformatics.

[36] Murakami S (2008). Multidrug efflux transporter, AcrB–the pumping mechanism. Curr. Opin. Struct. Biol..

[37] Schendel SL, Click EM, Webster RE, Cramer WA (1997). The TolA protein interacts with colicin E1 differently than with other group A colicins. J. Bacteriol..

[38] Levengood SK, Beyer WF, Webster RE (1991). TolA: a membrane protein involved in colicin uptake contains an extended helical region. Proc. Natl Acad. Sci. USA.

[39] Cascales E, Lloubes R, Sturgis JN (2001). The TolQ-TolR proteins energize TolA and share homologies with the flagellar motor proteins MotA-MotB. Mol. Microbiol..

[40] Lazzaroni JC, Dubuisson JF, Vianney A (2002). The Tol proteins of *Escherichia coli* and their involvement in the translocation of group A colicins. Biochimie.

[41] Schlor S, Schmidt A, Maier E, Benz R, Goebel W, Gentschev I (1997). In vivo and in vitro studies on interactions between the components of the hemolysin (HlyA) secretion machinery of *Escherichia coli*. Mol. Gen. Genet..

[42] Cox EC (1976). Bacterial mutator genes and the control of spontaneous mutation. Ann. Rev. Genet..

[43] Li XT, Thomason LC, Sawitzke JA, Costantino N, Court DL (2013). Positive and negative selection using the tetA-sacB cassette: recombineering and P1 transduction in *Escherichia coli*. Nucleic Acids Res..

