# Supplementary material for: Rational optimization of tolC as a powerful dual selectable marker for genome engineering
Source: Nucleic Acids Res. 2014 Jan 22;42(7):4779–90. doi: 10.1093/nar/gkt1374 (PMC3985617; doi:10.1093/nar/gkt1374)
Supplement: Supplementary Data [file supp_42_7_4779__index.html]

Rational optimization of tolC as a powerful dual selectable marker for genome engineering — Rational optimization of tolC as a powerful dual selectable marker for genome engineering — Supplementary Data 

# Rational optimization of *tolC* as a powerful dual selectable marker for genome engineering

## Supplementary Data

files

**Files in this Data Supplement:**

- Supplementary Data - pdf file
