# Supplementary material for: Rational optimization of tolC as a powerful dual selectable marker for genome engineering
Source: Nucleic Acids Res. 2014 Jan 22;42(7):4779–90. doi: 10.1093/nar/gkt1374 (PMC3985617; doi:10.1093/nar/gkt1374)
Supplement: Supplementary Data [file supp_gkt1374_nar-03197-met-k-2013-File006.pdf]

SUPPLEMENT

Rational Optimization of tolC as a Powerful Dual Selectable Marker for Genome Engineering

Christopher J. Gregg*, Marc J. Lajoie*†, Michael G. Napolitano‡, Joshua A. Mosberg†, Daniel B. Goodman, John Aach, Farren J. Isaacs[2], & George M. Church

Department of Genetics & Wyss Institute for Biologically Inspired Engineering,
Harvard Medical School, Boston, MA 02115,
†Program in Chemical Biology, Harvard University, Cambridge, MA 02138,
‡Biological and Biomedical Sciences, Harvard Medical School, Boston, MA 02115,
[2]Molecular, Cellular, Developmental & Systems Biology Institute, Yale University, New Haven, CT 06516, USA.

*The contributions of the first two authors are equal.

Address correspondence to:
George M Church
77 Avenue Louis Pasteur
New Research Building, Room 238
Harvard Medical School
Boston, MA 02115
Tel: 617-432-6219. Fax: 617-432-6513
gchurch@genetics.med.harvard.edu

Major Classification: Biological Sciences
Minor Classification: Genetics
Keywords: Microbial Genetics, *tolC*, Dual Selectable Marker, Recombineering

SUPPLEMENTAL RESULTS & DISCUSSION

Assessing the Dysfunctional Phenotype Associated with *tolC* Counter-selection Escape

To understand more about *tolC* counter-selection escape, we grew monocultures of EcNR2.*ΔtolC* and EcNR2.*tolC*$^+$ strains in colE1. Whereas EcNR2.Δ*tolC* were resistant to colE1 and grew without delay, 12/16 EcNR2.*tolC*$^+$ cultures exhibited growth after a significant delay. For example in one experiment, 4/6 EcNR2.*tolC*$^+$ replicates grew with a delay of 749.2±111.0 (mean±stdev) minutes compared to EcNR2.*ΔtolC* controls. Upon passaging these cultures into a 2nd counter-selection, the 4 EcNR2.*tolC*$^+$ replicates that had grown after a delay during the 1st colE1 selection now exhibited growth kinetics in colE1 that were indistinguishable from EcNR2.ΔtolC, implicating mutational escape rather than loss of colE1 activity (Fig. S3B).

To ascertain whether escape was due to mutation of the *tolC* coding region, we tested if *tolC* counter-selection escape clones could grow in SDS. Replica plating onto SDS demonstrated that 47.3%±10.0% (mean±stdev, n = 6) of escape clones were sensitive to SDS, and likely harbored inactivating mutations in the *tolC* coding region. While SDS-sensitive escape mutants are readily removed during any subsequent SDS selection, dysfunctional clones (~53% that are both SDS-resistant and colE1-resistant) can survive both selections and rapidly take over upon repeated cycles of SDS and colE1 selections. For example in a typical *tolC* CoS-MAGE cycle, the recombination frequency for the selectable oligo is ~10^{-2} – 10^{-1} recombinants per viable clone. In contrast, SDS-resistant/colE1-resistant cells pass both selections with a frequency of ~ 1; thus, dysfunctional escape clones rapidly take over a CoS-MAGE lineage, as the relative abundance between desired clones and escapees shifts by a factor of 10^1 – 10^2 per cycle. It is noteworthy that increasing the mole fraction of the selectable oligo had a statistically significant impact on delaying complete breakdown of the CoS-MAGE workflow (Fig. 1E), suggesting that improving recombination frequency through increasing oligo concentration significantly reduces the shift in relative abundance between desired clones and escapees from cycle to cycle. When escapees are a minor fraction of the population, they are more likely to be left behind upon dilution into fresh media.

To assess whether there is any growth phenotype associated with *tolC* counter-selection escape, we picked 6 SDS-resistant/colE1-resistant (dysfunctional) clones and 6 SDS-sensitive/colE1-resistant clones and monitored growth in colE1. These data are presented in Figure S3C as the time to reach OD_{600} = 0.4. SDS-sensitive/colE1-resistant clones (95 ± 22 min) grew indistinguishably from EcNR2.Δ*tolC* (115 ± 30 min., p > 0.05) in colE1, consistent with *tolC* inactivating mutations conferring the *tolC*$^-$ genotype. Dysfunctional clones that are SDS-resistant/colE1-resistant grew in colE1, but exhibited a significant delay (180 ± 79 min., *p = 0.013 vs. EcNR2.Δ*tolC*), consistent with the hypothesis that there are a variety of mutational escape strategies that may exhibit varying fitness when challenged with colE1.

Examples of Mutations that are Associated, but Unrelated to *tolC* Counter-selection Escape

Only one non-tolQRA mutation, a frameshift in *ogt* (XM_*ogt*_1398047_T_TC, 0.56±0.01), was putatively causal based on Normalized Culture Time. Three additional XM oligos targeting *recR* (0.73±0.02), *treB* (0.79±0.01),

and a putative intergenic site (nt 752378, 0.69±0.14) were also found to have borderline influence on counter-selection escape. These 4 coding regions have not been previously implicated in *tolC* function.

We further explored the nature of the SNV in the intergenic region at nt 752378, between *ybdG* and *gltA*, we hypothesized that polar effects may affect *ybdG* or *gltA*. Therefore, we tested whether either *ybdG* or *gltA* played a role in colE1 resistance by inactivating each gene with MAGE oligos *ybdG*_mut and *gltA*_mut. Notably, the *ybdG*_mut (1.146±0.265) and *gltA*_mut (1.292±0.471) mutations exhibited unrelated Normalized Culture Times, suggesting that any effect of XM_Intergenic_752378_T_G on colE1 resistance is not due to reducing/abolishing expression of surrounding coding regions.

To further study all 4 mutations, we performed additional biological replicates in a simpler experimental setup (Table S3) than the high-throughput validation (Table S2). These replicates were largely confirmatory, except for *ogt*, which was only weakly causal, consistent with a Borderline mutation. We then tested the mutant genotypes in isolation for their counter-selection escape phenotype. Whereas the previous validations have counter-selected from mixed populations containing the parental genotype, we used mascPCR to identify each of the four monoclonal mutations, then tested their colE1 phenotypes compared to EcNR2.Δ*tolC* and EcNR2.*tolC*$^+$ clonal controls. Under these conditions, the *ogt*, *recR*, and *treB* SNVs, as well as the intergenic SNV at nt 752378 grew with negative controls (Table S4), thus are likely unrelated to counter-selection escape.

Despite a cutoff for Borderline Causal as Normalized Culture Time < 1, we excluded XM_*Rep321e*_4294083_C_T from further analysis due to a relatively large error of measurement (0.933±0.116). Moreover, *rep321e* is a member of palindromic repeat elements between *gltP* and *yjcO*; thus, oligos targeting the mutation seen in our sequencing data set contain significant off-target homology to other repetitive elements, which obfuscates interpretation of the results.

Given previous literature implicating BtuB as the OM receptor for colE1 (1) and despite finding 3 unique mutations in BtuB (Table S5), we were surprised that *btuB* loss-of-function mutations were not classified as causal (Table S2). We duplicated *btuB* in a *tolQRA* duplicated strain (EcNR2.1255700::*tolQRA)* and found that 25/32 *btuB* duplication replicates escaped counter-selection, with extremely variable kinetics (OD_{600} = 0.4 at 1846.7 ± 662.9 minutes, mean ± stdev), whereras only 1/32 EcNR2.1255700::*tolQRA* parental replicates escaped (OD_{600} = 0.4 at 919.5 minutes). As a result, we did not pursue *btuB* duplication further; we hypothesize that increasing *btuB* copy number leads to off-target effects such as increasing the number of colE1 binding sites on the OM (1).

Multiple Nuclease Knockout Underlies Heat Shock-based Growth Phenotype Seen in Nuc5$^-$

We previously showed that knocking out a number of endogenous *E. coli* nucleases (*xonA*, *exoX*, *xseA*, *recJ* and *λexo*) in EcNR2.Nuc5$^-$ significantly improves AR frequency in CoS-MAGE (Fig. 1A-B, middle bar (2). However, EcNR2.Nuc5$^-$ exhibits a growth phenotype after recombination that resolves upon subsequent passage into fresh media (2). To explore this phenotype further, we isolated steps in the recombination workflow (42 C <u>Induction</u> of λ Red proteins, water <u>Wash</u>es on ice to reduce salt content, <u>Electroporation</u> with or

without oligos), then monitored growth to determine which step(s) underlie the phenotype. Figure S4A compares EcNR2 (top panel) with EcNR2.nuc5- (bottom panel), and shows a clear association of slower growth rate of EcNR2.nuc5^{-} with Induction (filled shapes). V_{max} quantifies this effect as ~50% longer doubling times in induced EcNR2.nuc5^{-} compared to induced EcNR2 controls (Fig. S4C). The association of the Induction (42 C heat shock for 15 min) with the EcNR2.nuc5- recovery phenotype suggests two possible explanations: (1) the inactivated nucleases may be important in the heat shock response; or (2) the nucleases may be important in minimizing the transcriptional burden of λ prophage expression (which contains genes beyond *exo*, *bet*, and *gam*). To exclude the former, we used MAGE to replace the entire λ prophage with a zeocin recistance cassette (Δλ::*zeoR*) in EcNR2 and EcNR2.nuc5^{-}. Then we performed similar subsets of the recombination workflow and monitored growth for the recovery phenotype (Fig. S4B, S4C). Interestingly, EcNR2.nuc5^{-}.Δλ::*zeoR* did not exhibit a re-growth phenotype, despite Induction, suggesting that the EcNR2.nuc5^{-} phenotype is likely due to expression of the λ Red operon.

To identify the inactivated nuclease(s) in EcNR2.nuc5^{-} that underlie this phenotype, we started with EcNR2.nuc4^{-} (λexo^{+}, so as to permit recombineering with dsDNA cassettes (3)) and separately reverted each of the four inactivated nuclease genes back to wild type using MAGE. After identifying monoclonal reversion mutants by mascPCR, we performed a multiplexed recombination (5.2 μM) and monitored re-growth. These data (Fig. 3A) confirm the recovery phenotype of EcNR2.nuc5^{-} (black line) with the V_{max} of EcNR2.nuc5^{-} (3.79±0.69, "Induced Washed Electroporated (Water)", Fig. S4D) approximately 50% lower than that of EcNR2 (6.61±0.47). Surprisingly, each of the 4 nuclease reversion strains (Fig. 3A) grew more like EcNR2 (blue line) than the EcNR2.nuc5^{-} parent (black line). Specifically, *recJ*$^{+}$ (orange, V_{max} = 7.69±0.79, p = 0.20 vs. EcNR2), *xseA*$^{+}$ (purple, 6.77±0.10, p = 0.70), *xonA*$^{+}$ (magenta, 5.48±0.42, p = 0.1), and *exoX*$^{+}$ (cyan, 5.21±0.81, p = 0.1) grew statistically similarly to EcNR2, and all grew faster than EcNR2.nuc5^{-} (**p = 0.004 vs. EcNR2). These results suggest that the re-growth phenotype in EcNR2.nuc5^{-} is not due to knockout of any single nuclease, and that reversion of any of the four inactivated nucleases in Nuc5^{-} is sufficient to ameliorate this phenotype. We chose to avoid reverting *xseA*, as its inactivation is associated with improved mutation inheritance at the 3' end of ssDNA oligos (2). We chose to avoid reverting *exoX* because this reversion strain exhibited the worst post-recombination recovery phenotype (Fig. S4D), although it was not statistically significantly slower than EcNR2 (p = 0.1). To decide between the remaining two nucleases (*recJ* and *xonA),* we used allele conversion in a single round of CoS-MAGE as criteria for our decision (Fig. 3B). Both the *recJ* (reported as Mean Allele Conversion ± SEM, 1.77±0.13) and the *xonA* (1.72±0.12) reversion strains yielded statistically similar mean allele conversions per clone (p = 0.16 by Kruskal-Wallis ANOVA) to each other and to the parent strain (1.55±0.13), suggesting that neither *recJ* reversion nor *xonA* reversion detracts from the improved MAGE performance seen in EcNR2.nuc5-. The *xonA* reversion strain exhibited a sporadic growth phenotype on carbenicillin-containing solid media, so we chose to instead revert *recJ* in MAGE-optimized strain EcM1.0.

***tolQRA* Duplication Enables Stable CoS-MAGE Cycling (continued from main text)**

Our computational model suggested that a CoS-MAGE lineage would incrementally approach a completely modified genotype over 10 cycles (Fig. 1C, Fig. S2), whereas our empirical data showed that the lineages moved much more erratically towards the completely modified genotype (Fig. 3CD). The model did not take into account the selection library size when extrapolating CoS-MAGE performance across multiple cycles, and these results indicate that genetic bottlenecks play an important role fixing minor variants in a mixed population. However, it is not obvious why the *tolC* counter-selection would consistently fix minor variants (clones harboring additional mutations to the parental genotype), rather than fixing the major variant (the parental genotype). It seems unlikely that counter-selection (colE1) on a *tolC*+ strain would have an intrinsically greater capacity to recombine oligos at neighboring loci than the selection (SDS) and it will be interesting to see if this observation is born out through future empirical data with larger sample sizes.

In terms of improvements over previous best practices, CoS-MAGE cycling in EcM2.0 yielded tenfold more changes per cycle than MAGE in EcNR2. This translates into less calendar time needed to attain a defined set of modifications. Although the total number of cycles needed to attain a defined set of modifications is an important metric for evaluating the power of MAGE vs. CoS-MAGE, another important consideration is the number of cell divisions required to achieve a desired genotype. Defective mismatch repair (*mutS::cat*) in EcNR2-based lineages is an important enabling technology for MAGE, but comes with an ~10-100-fold increased mutation frequency (primarily transition mutations) (4). For example, we used MAGE to change all 321 TAG stop codons to TAA synonyms genome-wide (5). During strain engineering, this strain acquired 355 off-target mutations over ~7340 doublings due to defective mismatch repair (5). One way to reduce off-target mutation frequency is to minimize the total number of cell divisions that a population will proceed through during genome editing. Although CoS-MAGE fixes ~6-fold more variants per cycle (Fig. 1AB), a CoS-MAGE cycle involves more cell divisions than a MAGE cycle, as the subpopulation of cells that recombine the selection oligo must undergo additional divisions during selection in order to repopulate the culture (only a subset of viable cells gain the selectable allele). By taking into account both workflows, we estimate that cells will go through 9 divisions in two cycles of MAGE (assuming a singleplex AR frequency of 10^{-1}), while cells will go through 25 to 31 (uncertainty due to estimates of AR frequency for the *tolC* inactivation/reversion oligos, estimated at 10^{-1} and 10^{-2}, respectively) divisions in two cycles of *tolC* CoS-MAGE. Thus, to completely modify 10 loci in a majority of the population, we estimate that MAGE will need ~860 cell divisions (90 cycles), whereas CoS-MAGE will need around 245 to 312 divisions (10 cycles). This represents a 64-72% reduction in the total number of cell divisions, and should reduce the off-target mutation burden in kind.

Calendar time is another important consideration for evaluating the power of MAGE vs. CoS-MAGE. To compare, we assume that the process cycle for MAGE is 1/8 day and that the process cycle for CoS-MAGE, which takes into account additional time needed for selection/counter-selection growth is 1/3 day. Based on these process cycles, we estimate that it will take 11.25 days, whereas CoS-MAGE will take 3.33

days to completely modify 10 loci in a majority of a population. This corresponds to a ~70% reduction in total time needed, similar to the savings in mutation burden above.

Appending the ssrA Degradation Tag to *tolC* Increases Protein Turnover, but Reduces Fitness in SDS. One suboptimal characteristic of TolC is its slow turnover time from the OM, which necessitates long recovery times between the inactivation recombination and the corresponding colE1 counter-selection. To increase TolC turnover rate, we appended the 11 amino acid ssrA tag (6,7) to the C-terminus of the *tolC* coding sequence. To assess and optimize emergent phenotypes associated with ssrA tagging *tolC*, we used several ssrA tag variants reported to effect different protein half-lives. Tested variants included ssrA^{FULL} (AANDENYALAA (6)), ssrA^{INT1} (AANDENYALAS), ssrA^{INT2} (AANDENYADAA), and ssrA^{WEAK} (AANDENYADAS (6)). To assess whether ssrA-tagging *tolC* supported shorter recovery times prior to colE1 selection, we performed recombinations using oligo *tolC*-r.null_mut on EcNR2 harboring the 4 *tolC_ssrA* variants, then inoculated colE1 selections at 15, 45, 90, and 180 minutes, as well as after the standard 7 hour recovery. Only *tolC*_ssrA^{FULL} supported successful colE1 selections at shorter (< 7 hrs.) recovery times, including 15 minutes (Fig. S5A, top panel, dark red trace), consistent with the 1 minute half-life previously observed for GFP_ssrA^{FULL} (6), whereas all variants grew after the full 7 hour recovery. The *tolC*_ssrA^{INT1}, *tolC*_ssrA^{INT2}, and *tolC*_ssrA^{WEAK} variants lacked any apparent intermediate phenotype and did not grow in colE1 after any recovery time less than 7 hours (Fig. S5A). We then tested the SDS resistance of ssrA-tagged *tolC* cassettes and found that *tolC*_ssrA^{FULL} exhibited reduced fitness at concentrations of 0.01% w/v SDS and above (Fig. S5B). Untagged *tolC*, as well as the weaker ssrA tagged *tolC* variants (*tolC*_ssrA^{INT1} and *tolC*_ssrA^{INT2}, not shown), exhibited no apparent growth phenotype in up to 0.08% w/v SDS. The growth phenotype in SDS was problematic, as the ssrA^{FULL} tag consistently mutated during normal culture in SDS, making it difficult to manage the complex selection pressures associated with ssrA tagging *tolC*.

We attempted to optimize expression of the *tolC*_ssrA^{FULL} cassette by using MAGE to explore promoter mutations that would increase expression and offset increased turnover. Although we optimized the promoter toward the canonical *E. coli* σ^{70} promoter, we were unable to alleviate the observed SDS phenotype. We also attempted to mutate the ssrA^{FULL} tag with degenerate MAGE oligos to randomize the 11 amino acid ssrA sequence. After each round of MAGE, we selected against the growth phenotype using 1x SDS. After 6 rounds of MAGE, we performed a *tolC*-r.null_mut recombination, followed by a colE1 selection after a 30 minute recovery to ensure tag function. Sequencing showed that the tag was surprisingly resistant to mutation, and subsequent SDS phenotyping showed that the growth phenotype remained. These results suggested that the window between the non-selective dose and the maximum tolerated efficacious dose of SDS was too small for *tolC*_ssrA^{FULL} strains (~4 fold, based on Fig. S5B, compared to ≥ 64-fold for untagged *tolC*). One potential explanation for our lack of success is that different proteases are responsible for degrading ssrA-tagged proteins in the cytosol (*clpA*, *clpX*, *lon*, *etc.*) versus the OM/PP (*prc*). (8). Our results are consistent with *prc* requiring the C-terminus of the ssrA tag to end in LAA (9), which was only the case for ssrA^{FULL}. Thus, it

should be recognized that different proteases prefer distinct, yet overlapping specificities within the ssrA tag sequence, making modular application of some ssrA tag variants problematic.

Validating Colicin Agar Plates to Quantify *tolC* Counter-selection Escape Frequency

We generated vancomycin ($LB^{L}CV$), colE1 ($LB^{L}CCo$), and colE1/vancomycin ($LB^{L}CCoV$) agar plates as described in the **Methods**. To validate these plates, we plated EcNR2.tolC^{+} onto $LB^{L}CV$ (2E3 cells), $LB^{L}CCo$ (1E7 cells), and $LB^{L}CCoV$ (1E7 cells). These inocula were chosen to yield a countable number of clones on the given media. As shown in Figure S6A, EcNR2.*tolC*$^{+}$ readily escapes selection on the $LB^{L}CV$ plates (8.3E-2 ± 1E-1), while the $LB^{L}CCo$ plates are significantly more stringent (3.4E-5 ± 1.7E-5). As expected, the colE1/vancomycin ($LB^{L}CCoV$) plates are significantly more stringent (4.5E-8 ± 9E-9) than colE1 ($LB^{L}CCo$) alone. To demonstrate that these plates can be used for *tolC* counter-selection, we streaked 10^{6} EcNR2.*tolC*$^{+}$ and EcNR2.*ΔtolC* cells onto $LB^{L}CV$, $LB^{L}CCo$, and $LB^{L}CCoV$ plates. As shown in Figure S6B, EcNR2.ΔtolC grows without apparent phenotype, while EcNR2.*tolC*$^{+}$ yields markedly fewer colonies despite streaking the same number of cells. As in Figure S6A, we observed more EcNR2.*tolC*$^{+}$ growth on $LB^{L}CV$ than $LB^{L}CCo$, while $LB^{L}CCoV$ permitted the least growth. This again suggests that the $LB^{L}CCoV$ plates are more stringent than either counter-selection agent used alone.

To test whether these plates are bactericidal rather than bacteriostatic, we performed a CoS-MAGE cycle on EcM2.0.*tolC*$^{+}$ cells using 1 μM tolC-r.null_mut, recovered for 7 hours, then plated the recovery cultures onto $LB^{L}CCoV$ plates to counter-select. After overnight incubation, we picked 96 clones from the $LB^{L}CCoV$ plates into LB^{L} + Carb and LB^{L} + Carb + SDS. As shown in Fig. S6C, these clones grew normally in LB^{L} + Carb, but none grew in LB^{L} + Carb + SDS. This demonstrates that the $LB^{L}CCoV$ plates successfully kill the parental (*tolC*$^{+}$) genotype, and indicates that clones picked from the plate-based counter-selection do not carry viable *tolC*$^{+}$ contamination.

Supplemental Discussion of Future Candidate Dual Selectable Markers for Optimization

It will be informative to apply this unbiased workflow to diagnose selection escape with other dual selectable markers, such as the sugar utilization kinases (*malK*, *galK*, etc.) (10), the multi-functional tetracycline efflux protein *tetA* (11), Herpes simplex virus thymidine kinase (hsvTK) and other thymidine-based markers (*thyA*). The sugar utilization kinases are required for growth in media containing the respective sugar as a sole carbon source, while rendering the cell sensitive to toxic analogues. Selections based on sugar utilization must be performed in minimal media to maintain selection stringency, thus they require significant growth time. Of note, *tetA* confers tetracycline resistance through efflux, but also confers sensitivity to Ni- and Cd-salts. The striking similarity of the selection mechanisms associated with the *tetA* efflux protein and those of *tolC* suggest that *tetA* may be amenable to the workflow described here to make *tetA* a more robust dual-selectable marker.

Herpes thymidine kinase is an interesting case because selective pressure for selection escape would lead to mutations in off-target alleles, whereas selective pressure for counter-selection escape would lead to mutations in the marker itself (12). In the selection, the marker rescues thymidine deficiency in the context of inhibiting endogenous thymidine metabolism (*thyA*). In a scenario that is analogous to *tolC*, one would predict loss-of-function mutations (that interfere with *thyA* inhibition) would permit selection escape, and further predict that the selection escape frequency would be reduced by duplicating *thyA*. On the other hand, the counter-selection exploits the substrate promiscuity of the marker to incorporate deleterious nucleoside analogues into the genome. Thus, one would predict selective pressure for deleterious mutations within the marker coding region itself. It remains to be seen how biology escapes these selection mechanisms and how, if at all, we are able to engineer a strain to avoid that escape.

SUPPLEMENTAL METHODS

Lambda Red Recombinations, MAGE, & CoS-MAGE

Lambda Red recombineering is the basis for MAGE and CoS-MAGE, which were carried out as described previously (2,13,14). Overnight liquid culture was passaged 1:100 into fresh LB^L and grown in a rotator drum at 30 °C and 150 rpm. When OD_{600} reached 0.4-0.6, cultures were heat induced in a shaking water bath at 42 °C and 300 rpm for 15 minutes. Cultures were placed on ice for 5 minutes and the remaining preparation was performed at 4 °C: 1 mL of culture was washed twice in 1 mL of dH_2O by centrifugation at 16,100 rcf for 20 seconds. After the final wash, the supernatant was removed, and the pellet was resuspended in 50 µL of dH_2O containing the DNA for recombination. Resuspended cell pellets containing the recombinogenic DNA were electroporated in a 0.1 cm gap cuvette (Biorad) using a Gene Pulser (Biorad) at 1.78 kV, 25 µF, 200 Ω. Electroporation time constants for properly washed samples ranged from 4.6 to 4.8 seconds. Electroporated cells were immediately recovered in 3 mL LB^L in a rotator drum at 30 °C for at least 1 hour (selectable markers), 3 hours (non-selectable alleles), or 7 hours (*tolC* counter-selections) before selection, plating, or further recombinations.

Notes on *tolC*-based Selections

The TolC protein is a multi-functional pore that provides a route for efflux of a wide variety of small molecules, salts, and macromolecules. As such, loss of *tolC* leads to a pleiotropic phenotype, including impaired cell division and morphology, and a broad sensitivity to environmental agents, including antibiotics and common metabolites (15). Although the *tolC* selection used in this work is based on efflux of a membrane compromising detergent (SDS), we also noted *tolC*-dependent sensitivity to antibiotics with intracellular mechanisms of action. For example, the mismatch repair gene *mutS* was replaced with chloramphenicol (Cm) acetyl-transferase (*mutS::cat*) in EcNR2-based strains. Despite the presence of the resistance gene, EcNR2.*tolC*$^-$ exhibits reduced fitness in Cm with respect to EcNR2.*tolC*$^+$ (Fig. S1D). As the *tolC*-dependent Cm phenotype suggests that *tolC* plays a role in Cm efflux regardless of the presence of *cat*, we perform *tolC* selections in SDS plus Cm to take advantage of added selection robustness. A similar *tolC*-dependent phenotype has been observed for kanamycin, X-gal/IPTG, and McConkey media, agents that also gain access to the intracellular space for their respective actions. It is important to consider these observations when designing experimental protocols using *tolC* as a selectable marker, especially in strains bearing many selectable markers where antibiotic use can cause unexpected *tolC* selection/counter-selection failure.

Although published protocols (16) call for a single 5 hour recovery before inoculating the counter-selection using ~$2*10^7$ stationary phase cells, we have observed that inoculating the counter-selection using ~$5*10^5$ early mid-log cells is crucial for optimal counter-selection performance, possibly by allowing additional time for TolC turnover.

We used *tolC*_null_mut/*tolC*_null_revert oligos to inactivate/re-activate *tolC* genes located on the plus strand of replichore 1 and on the minus strand of replichore 2, while we used *tolC*-r_null_mut/*tolC*-r_null_revert oligos to inactivate/re-activate *tolC* genes located on the minus strand of replichore 1 and the plus strand of replichore 2 (including the endogenous MG1655 *tolC*). Sequences for these oligos are given in Table S6.

CoS-MAGE Modeling

The input data for CoS-MAGE modeling were the genotypes of the 10 targeted loci from 92 clones of a population of EcNR2.nuc5-.*dnaG*_Q576A cells that had been subjected to one cycle of CoS-MAGE. Genotypes were represented as binary vectors $G_j = (\gamma_{j,1}, \gamma_{j,2}, \dots, \gamma_{j,10})$, where $\gamma_{j,i} = 1$ if locus i $(i = 1,2,\dots,10)$ was MAGE-mutated and $\gamma_{j,i} = 0$ if locus i was not mutated. The complete space of possible genotypes comprises 2^{10}=1024 vectors, which defines the range of index j. Our models were designed to compute probability distributions $P(G_j, n)$ for the entire space of 1024 genotypes for each of a series of 15 consecutive CoS-MAGE cycles $n = 1,2,\dots,15$. The 92 genotypes from the clone data were used to define the probabilities of allele replacement patterns affected by each individual MAGE cycle. Different versions of the model were constructed to either take into account or ignore correlations in allele replacement seen between alleles in the empirical data. *For the correlated model* (Fig. S2): The genotype distribution of the first CoS-MAGE cycle $P(G_j, 1)$ was taken to be the actual frequency distribution of the genotypes found in the 92 experimental clones. Then, for cycle n > 1, for 10,000 random simulations, a genotype was randomly chosen using the probability distribution $P(G_j, n-1)$, and combined with an allele replacement pattern randomly chosen from probability distribution $P(G_k, 1)$ from the first cycle. Here "combination" consisted of taking the union $G^* = U(G_j, G_k)$ of all allele replacements in the genotype and the allele replacement pattern, i.e., $U(G_j, G_k) = \left(\max\left(\gamma_{j,1}, \gamma_{k,1}\right), \max\left(\gamma_{j,2}, \gamma_{k,2}\right), \dots, \max(\gamma_{j,10}, \gamma_{k,10})\right)$. $P(G_j, n)$ was then computed as the frequency distribution of the 10,000 union genotypes G^* obtained from these simulations. By basing the incremental effect of each cycle on the 96 experimental genotypes, this model propagated the effect of all correlations between allele replacements inherent in the actual data throughout all cycles. *For the uncorrelated model* (Fig. 1C): Individual allele replacement frequencies AR_i for $i = 1,2,\dots,10$ were computed for each of the 10 loci from the genotype frequencies in the actual data. Then, for each cycle, the probability of obtaining each of the 1024 possible genotypes was computed through an exact calculation. Specifically, for the first cycle, $P(G_j, 1)$ was computed as $\prod_{i:\gamma_{j,i}=1} AR_i \cdot \prod_{i:\gamma_{j,i}=0} (1 - AR_i)$ for all 1024 genotypes G_j. Then, for cycle n > 1, an iteration was made through all 1024^2 pairs (G_j, G_k) of genotypes, and for each pair the probability $P(G_j, n-1)P(G_k, 1)$ was aggregated to the value $P(U(G_j, G_k), n)$. By computing genotype probabilities from individual AR_i frequencies only, this model ignored any effect of correlations between the allele replacements in the actual data. Finally, for both models, the distribution of numbers of replaced alleles

for each cycle was computed from the 1024 $P(G_j, n)$ values by aggregating these probabilities to each of the eleven possible values 0, 1, …, 10 of the sums $\sum_{i=1,..,10} \gamma_{j,i}$.

Only subtle differences can be seen between the uncorrelated (Fig. 1C) and correlated (Fig. S2) distributions: Broadly speaking, the uncorrelated distributions that treat each allele as independent tend to be narrower and more regular than the distributions that take correlations into account. The two sets of distributions approach each other after many cycles, suggesting that the effect of correlations diminishes as cycles increase. This is reasonable because MAGE edits accumulate so that as cycles increase, there is less opportunity for the correlated and uncorrelated processes to yield different results.

High-throughput Sequencing of *tolC* Counter-selection Escape Clones

tolC counter-selection escape clones (n=96) were grown up overnight in 1 mL LB^L supplemented with SDS and colE1. Genomic DNA was prepared by washing confluent cultures in 0.5 mL 1x TE buffer (10 mM Tris, 1 mM EDTA, pH 8.0) and spinning them at 3,000 rpm for 5 minutes. Pellets were resuspended in 0.5 mL TE plus 1 µg RNase A (Qiagen), transferred to a Covaris microTUBE plate and sheared overnight using a Covaris E210 Ultrasonicator, duty cycle of 10%, intensity of 5, 200 cycles/burst, 500 seconds/well, and a vertical offset of -4.0 mm. Sheared genomic DNA was size selected using a 2-step SPRI purification where 130 µL of sheared DNA plus 70 µL dH_2O was mixed with 0.275x SPRI beads, which preferentially captures large genomic fragments. The supernatant was then transferred to a second SPRI purification using 0.85x SPRI beads, which preferentially captures fragments larger than 200 bp. Captured, sheared DNA was washed twice in fresh 70% ethanol, dried for 20 minutes, and eluted using 20 µL of 0.1x TE. Sheared fragments were blunt end repaired using the Quick Blunting Kit (New England Biolabs) using ¼ of the recommended enzyme, but otherwise according to the manufacturer's protocols. A blunting reaction was carried out at 12 °C (20 min) followed by 37 °C (15 min). Blunted DNA fragments were SPRI purified using 2x beads, eluted in 28.8 µL of 200 µM Illumina sequencing adapters, and added to 31.2 µL barcoded ligation reactions, according to the Quick Ligation Kit (New England Biolabs) recommended protocol. Barcoded, Illumina-compatible adapters were prepared for this ligation by annealing complimentary oligos in stoichiometric ratios and incubating at 95 °C for 5 minutes followed by a ramping incubation down to 37 °C using 0.2 °C/sec (*barcoded P5-short* with *barcoded P5-short-compl* and universal *P7-short-compl* with *universal P7-short*). After annealing, the appropriate P5 and P7 adapters were mixed in preparation for the ligation. At this point, each monoclonal sequencing library was labeled with its own 6bp barcode. Our barcoding strategy can be found in Table S6. Adapter-ligated DNA was then SPRI purified using 1.6x beads and eluted in 40 µL of 0.1x TE. Purified adapter-ligated DNA fragments were then filled in using Bst DNA polymerase (New England Biolabs) at 37 °C for 15 minutes. Filled-in adapter-ligated fragments were SPRI purified using 1.6x beads and eluted using 30 µL 0.1x TE into a 50 µL adapter-ligated DNA fragment enrichment PCR using Phusion High Fidelity polymerase (New England Biolabs) and primers *Sol-P5-PCR* & *Sol-P7-PCR*. The reaction was carried out by first denaturing at 98 °C (2 minutes), followed by 10 cycles of 98 °C (10 seconds), 62 °C (20 seconds), 72 °C (20 seconds) and a final

extension at 72 °C (5 minutes). PCR products were SPRI purified using 1.6x beads and eluted in 50 µL of 0.1x TE and stored at -80 °C. Monoclonal genomic libraries were quantitatively controlled for pooling in a 96 well, DyNAmo SYBR Green qPCR reaction (New England Biolabs) using the *Sol-P5-PCR* & *Sol-P7-PCR* primer set and 1 µL of 1E4-fold diluted template. The qPCR reaction was carried out by first denaturing at 95 °C (15 minutes) followed by 40 cycles of 95 °C (10 seconds), 55 °C (45 seconds), 72 °C (30 seconds) on an Opticon 2 thermocycler (MJ Research). Equal concentrations of each monoclonal library were pooled and the size range of fragments was checked on a 2100 Bioanalyzer (Agilent Technologies) prior to high-throughput sequencing on an Illumina HiSeq2000.

Assessing Causality of Alleles Identified via Sequencing

To determine whether the alleles identified by sequencing resulted in the dysfunctional *tolC* phenotype, we designed MAGE oligos to generate the 55 most abundant mutations observed in our data set (XM oligos). We also designed MAGE oligos to knock out the 20 most frequently mutated coding regions, via premature stop codons placed within the first 150 bp of the start codon (cvrALL oligos). There are a number of genes which only had 1 or 2 unique mutations (Fig. 2B, bottom panel, columns to right of break in x-axis), but showed up in 5+ genomes (Fig. 2B, top panel, columns to right of break in x-axis). These include (# of unique coding mutations/# of genomes bearing mutation): *fixC* (2/5), *ydrM* (2/11), *frmR* (1/11), *hyfR* (1/6), *rep321e* (1/8), *rnhA* (1/6), *wza* (1/12), *ybaE* (1/5), and *yjgf* (1/6). Although the number of unique mutations affecting these coding regions does not suggest a common mutation underlying counter-selection escape, it is difficult to rule out causality given the non-trivial number of independent clones exhibiting these mutations; thus, we included oligos to encode the mutations found in *ydrM*, *frmR*, *rep321e*, and *wza*.

These oligos were recombined into EcNR2.*tolC*$^+$, recovered overnight, and then selected in colE1 on a Spectramax M2 Plate Reader. These recombinations and selections were performed in duplicate. To quantify relative causality, we included multiple controls (Table 1, dark blue shaded rows), such as EcNR2.*tolC*$^+$ recombined with 1 µM tolC-r.null_mut (positive control) or with water (negative control), which were used to calculate Normalized Culture Time according to the following formula, $(t^x-t^{pos})/(t^{neg}-t^{pos})$, where t^{pos} is the average culture time to OD_{600} = 0.4 for the positive control replicates, t^{neg} is the average culture time for negative control replicates, and t^x is the culture time to OD_{600} = 0.4 for cultures recombined with oligo X. As an additional control, the cvrALL_*tolC*_3 oligo introduces a premature stop codon into the beginning of the *tolC* coding region, and is predicted to lead to colE1 resistance with similar kinetics as the *tolC*-r.null_mut positive controls.

Normalized Culture Time was defined as 0 for EcNR2.*tolC*$^+$ recombined with *tolC*-r.null_mut and 1 for EcNR2.*tolC*$^+$ recombined with water, which enables direct replicate-to-replicate comparison. Causal mutations were defined as those exhibiting 0 < Normalized Culture Time < 0.6. This cut-off was chosen to have a high confidence of covering all obvious candidate mutations/genes. Borderline mutations were defined as 0.6 ≤ Normalized Culture Time < 1.0, whereas unrelated mutations exhibited Normalized Culture Time ≥ 1.0. While

this approach enables high-throughput assessment of causal mutations, Normalized Culture Time is affected by both mutation causality and oligo recombination frequency, which is known to vary significantly from oligo to oligo (13). While oligos that resulted in Normalized Culture Times < 0 are likely due to oligos that are more recombinogenic (not "more causal") than tolC.null_mut, this metric provides a quantitative strategy for identifying candidate alleles that can be easily validated synthetically.

SUPPLEMENTAL REFERENCES

1. Lazzaroni, J.C., Dubuisson, J.F. and Vianney, A. (2002) The Tol proteins of Escherichia coli and their involvement in the translocation of group A colicins. *Biochimie*, **84**, 391-397.
2. Mosberg, J.A., Gregg, C.J., Lajoie, M.J., Wang, H.H. and Church, G.M. (2012) Improving lambda red genome engineering in Escherichia coli via rational removal of endogenous nucleases. *PloS one*, **7**, e44638.
3. Mosberg, J.A., Lajoie, M.J. and Church, G.M. (2010) Lambda red recombineering in Escherichia coli occurs through a fully single-stranded intermediate. *Genetics*, **186**, 791-799.
4. Cox, E.C. (1976) Bacterial mutator genes and the control of spontaneous mutation. *Annual review of genetics*, **10**, 135-156.
5. Lajoie MJ, R.A., Goodman DB, Aerni HR, Haimovich AD, Mercer JA, Wang HH, Carr PA, Mosberg JA, Rohland N, Schultz PG, Jacobson JM, Rinehart J, Church GM, Isaacs FI. (2013) Genomically Recoded Organisms Impart New Biological Functions. *Science*.
6. Andersen, J.B., Sternberg, C., Poulsen, L.K., Bjorn, S.P., Givskov, M. and Molin, S. (1998) New unstable variants of green fluorescent protein for studies of transient gene expression in bacteria. *Applied and environmental microbiology*, **64**, 2240-2246.
7. McGinness, K.E., Baker, T.A. and Sauer, R.T. (2006) Engineering controllable protein degradation. *Molecular cell*, **22**, 701-707.
8. Moore, S.D. and Sauer, R.T. (2007) The tmRNA system for translational surveillance and ribosome rescue. *Annual review of biochemistry*, **76**, 101-124.
9. Flynn, J.M., Levchenko, I., Seidel, M., Wickner, S.H., Sauer, R.T. and Baker, T.A. (2001) Overlapping recognition determinants within the ssrA degradation tag allow modulation of proteolysis. *Proceedings of the National Academy of Sciences of the United States of America*, **98**, 10584-10589.
10. Warming, S., Costantino, N., Court, D.L., Jenkins, N.A. and Copeland, N.G. (2005) Simple and highly efficient BAC recombineering using galK selection. *Nucleic acids research*, **33**, e36.
11. Stavropoulos, T.A. and Strathdee, C.A. (2000) Expression of the tetA(C) tetracycline efflux pump in Escherichia coli confers osmotic sensitivity. *FEMS Microbiol Lett*, **190**, 147-150.
12. Tashiro, Y., Fukutomi, H., Terakubo, K., Saito, K. and Umeno, D. (2011) A nucleoside kinase as a dual selector for genetic switches and circuits. *Nucleic acids research*, **39**, e12.
13. Wang, H.H., Isaacs, F.J., Carr, P.A., Sun, Z.Z., Xu, G., Forest, C.R. and Church, G.M. (2009) Programming cells by multiplex genome engineering and accelerated evolution. *Nature*, **460**, 894-898.
14. Lajoie, M.J., Gregg, C.J., Mosberg, J.A., Washington, G.C. and Church, G.M. (2012) Manipulating replisome dynamics to enhance lambda Red-mediated multiplex genome engineering. *Nucleic acids research*, **40**, e170.
15. Koronakis, V., Eswaran, J. and Hughes, C. (2004) Structure and function of TolC: the bacterial exit duct for proteins and drugs. *Annual review of biochemistry*, **73**, 467-489.
16. DeVito, J.A. (2008) Recombineering with tolC as a selectable/counter-selectable marker: remodeling the rRNA operons of Escherichia coli. *Nucleic acids research*, **36**, e4.
17. Koronakis, V., Sharff, A., Koronakis, E., Luisi, B. and Hughes, C. (2000) Crystal structure of the bacterial membrane protein TolC central to multidrug efflux and protein export. *Nature*, **405**, 914-919.

SUPPLEMENTAL TABLES AND LEGENDS

Table S1. Fixed Variants in EcNR2.

Gene Name	Bio type	Effect	Sequence Context: Codon (Old / New)	Sequence Context: Amino Acid (Old / New)	Codon #	Coding Size (bp)	Chr. Pos.: MG1655	Chr. Pos.: Bayes	Base Call: Reference	Base Call: Deviation	# of Mutations in (96) Set
ylbE_1	mRNA	Synonymous	gaA / gaG	E / E	38	260	547694	547694	A	G	96
ylbE_1	mRNA	FRAME_SHIFT (Stop Lost)	cAg ggg aag tga / cAG ggg gaa gtg a…		84	260	547831	547831	A	AG	96
lhr	mRNA	NON_SYNONYMOUS_CODING	gGc / gAc	G / D	229	4617	1727796	1737003	G	A	96
yebT	mRNA	NON_SYNONYMOUS_CODING	Gaa / Aaa	E / K	89	2634	1915798	1925005	G	A	96
molR2	mRNA	NON_SYNONYMOUS_pseudo	taT / taC	Y / Y	286	1938	2196289	2205496	T	C	96
bglX	mRNA	SYNONYMOUS_CODING	Ctg / Ttg	L / L	294	2298	2219132	2228339	G	A	96
emrA	mRNA	SYNONYMOUS_CODING	gtA / gtG	V / V	231	1173	2810141	2819348	A	G	96
mutS::cat	mRNA							2865173	C	T	96
yhcF	mRNA	NON_SYNONYMOUS_CODING	Gga / Aga	G / R	10	717	3364975	3372727	G	A	96
dctA	mRNA	NON_SYNONYMOUS_CODING	cTg / cCg		221	1287	3680809	3688561	T	C	96
rbsA	mRNA	SYNONYMOUS_CODING	ggC / ggT	G / G	288	1506	3932664	3940416	C	T	96
yjgL	mRNA	FRAME_SHIFT	gcC ggg ggg cag … / gcC Ggg ggg gca g…		40	1815	4473579	4481331	C	CG	96
ylbE_1	mRNA	Synonymous	gaA / gaG	E / E	38	260	547694	547694	A	G	96
bioF								809843	T	C	96
bioF								810574	G	A	96
bioC								810793	A	T	96
uvrB	mRNA	NON_SYNONYMOUS_CODING	gAt / gGt	D / G	338	2022	813761	813761	A	G	96
moA	mRNA	SYNONYMOUS_CODING	gaT / gaC	D / D	50	990	816416	816416	T	C	96
fucK	mRNA	NON_SYNONYMOUS_CODING	gAc / gGc	D / G	122	1449	2935824	2943576	A	G	95
ppiC	Non-coding	VARIANT	tccgggcCtcgcag / tccgggcTtcgcag	121 bp upstream of atg			3957957	3965709	C	T	95
paaG	mRNA	SYNONYMOUS_CODING	gcA / gcG	A / A	118	789	1456641	1465848	A	G	93
flhD	Non-coding	VARIANT	attacCCatttatgt / attacATatttatgt	355 bp upstream of atg			1985734	1985734	GG	TA	53

Table S1. Fixed Variants in EcNR2. Deep sequencing of the dysfunctional clone set revealed a number of fixed polymorphisms that deviated from the *E. coli* K12 MG1655 reference genome. Since this clone set was derived from modified MG1655-based strain EcNR2, we report these polymorphisms as specific to that lineage and not related to mutations arising due to colE1 killing pressure.

Table S2. Assessing Causality of Alleles Identified via High Throughput Sequencing.

Mutation ID	Normalized Lagtime		Normalized t @ V_{max}		% Change Vmax	
	ave	st. dev.	ave	st. dev.	ave	st. dev.
water (E2.*tolC*-)	-0.480	0.007	0.183	0.024	0.578	10.699
water (E2.*tolC*-)	-0.480	0.007	0.175	0.012	6.823	18.912
XM_*tolA*_776642_GGCAA_G	-0.129	0.014	0.233	0.040	-14.703	0.525
tolC-r.null_mut (5 μM)	-0.074	0.007	0.251	0.037	4.213	17.074
XM_*tolR*_775139_A_G	-0.031	0.012	0.276	0.042	-15.359	4.216
tolC-r.null_mut	-0.012	0.016	0.261	0.031	1.780	10.842
XM_*tolR*_775339_C_T	0.007	0.121	0.315	0.112	-32.639	16.215
cvrALL_*tolC*_3	0.010	0.014	0.260	0.040	-0.392	5.621
tolC-r null_mut (2μM)	0.021	0.002	0.276	0.033	4.657	12.762
XM_*tolA*_776256_AGG_A	0.127	0.016	0.292	0.050	-16.395	5.221
XM_*tolA*_775912_G_GA	0.148	0.014	0.297	0.043	-16.507	9.065
XM_*tolC*_3176840_T_C	0.208	0.098	0.320	0.050	2.927	8.759
cvrALL_*tolQ*_14	0.219	0.114	0.404	0.135	-36.997	15.930
*tolC*_3176564_A_G	0.244	0.019	0.315	0.057	0.101	1.699
XM_*tolA*_775823_C_T	0.256	0.003	0.322	0.039	-11.102	3.646
*tolC*_3177340_T_C	0.256	0.003	0.327	0.040	2.994	0.089
*tolC*_3176753_A_G	0.289	0.016	0.331	0.042	3.863	3.289
*tolC*_3176813_A_G	0.299	0.002	0.322	0.063	-0.932	3.622
*tolC*_3176270_A_G	0.351	0.007	0.337	0.065	3.589	4.137
XM_*tolQ*_774584_A_AG	0.361	0.175	0.453	0.106	-36.350	4.793
*tolC*_3176582_A_G	0.416	0.000	0.351	0.069	-0.444	4.313
XM_*tolQ*_774558_A_ACT	0.458	0.200	0.455	0.118	-33.578	2.725
cvrALL_*tolR*_8	0.473	0.081	0.379	0.084	-26.490	15.351
XM_ogt_1398047_T_TC	0.564	0.014	0.383	0.071	-15.520	4.904
XM_*tolA*_776380_CA_CAA,C	0.566	0.016	0.387	0.057	-8.230	2.886
XM_Intergenic_752378_T_G	0.689	0.135	0.442	0.105	-24.249	12.642
XM_recR_493693_C_T	0.726	0.018	0.430	0.068	-14.219	5.829
cvrALL_*tolA*_23	0.731	0.109	0.432	0.081	-8.875	1.035
cvrALL_treB_3	0.788	0.005	0.425	0.082	4.296	4.519
XM_*tolA*_776493_C_CG	0.830	0.031	0.433	0.078	-9.253	0.734
XM_*tolQ*_774693_A_AG	0.904	0.158	0.503	0.074	7.594	42.236
XM_*tolA*_776113_GA_G	0.907	0.023	0.458	0.074	-4.623	2.215
XM_Rep321e_4294083_C_T	0.933	0.116	0.496	0.123	-18.538	15.160
water	0.981	0.016	0.473	0.085	0.755	3.538
XM_fumC_1683605_GC_G	0.994	0.062	0.490	0.084	-3.916	1.833
cvrALL_uvrB_5	1.006	0.079	0.475	0.113	-1.313	0.935
XM_yfiH_2732557_T_TC	1.026	0.051	0.475	0.113	-1.798	1.604
XM_hyfB_2600077_G_GC	1.026	0.051	0.475	0.113	-5.815	5.136
XM_ftsZ_105469_A_T	1.034	0.006	0.496	0.083	-6.591	0.353
water	1.034	0.006	0.477	0.095	-2.065	1.686
XM_eaeH_313960_T_TC	1.047	0.053	0.494	0.086	-5.008	0.289

cvrALL_lhr_5		1.049	0.083	0.505	0.095	-0.007	3.695
XM_ydeK_1592317_T_TC		1.059	0.069	0.487	0.105	-0.159	2.078
XM_insE5_2168234_T_C		1.064	0.036	0.499	0.072	-6.096	0.592
XM_tynA_1449056_G_A		1.067	0.025	0.499	0.087	-0.824	1.626
XM_polA_4045942_A_AC		1.067	0.025	0.502	0.082	0.379	0.996
XM_hyfR_2610011_G_A		1.069	0.055	0.493	0.104	-2.719	3.525
XM_iraM_1211303_A_AC		1.069	0.055	0.484	0.116	-4.783	1.527
cvrALL_malZ_3		1.069	0.055	0.497	0.099	-3.268	0.936
cvrALL_recE_3		1.082	0.102	0.512	0.092	0.055	0.539
XM_leuV_4604154_A_AG		1.088	0.027	0.489	0.102	-0.433	1.230
cvrALL_*btuB*_3		1.088	0.027	0.504	0.080	-6.858	0.436
XM_ydiM_1769931_G_A		1.097	0.017	0.494	0.086	-4.461	0.781
XM_yjgF_4468858_T_A		1.097	0.017	0.496	0.083	-2.059	2.616
XM_elfC_1000951_C_CG		1.097	0.017	0.505	0.071	-2.453	0.677
XM_yehA_2186104_G_GC		1.097	0.017	0.508	0.075	0.815	1.614
XM_fepE_617633_C_CG		1.105	0.062	0.506	0.062	-4.184	1.915
XM_ynfE_1658003_G_A		1.107	0.031	0.508	0.075	-2.121	1.147
XM_yifK_3979054_G_GC		1.108	0.001	0.493	0.104	-4.629	1.285
XM_ycgB_1235495_A_AG		1.118	0.015	0.510	0.079	2.457	3.936
XM_ilvA_3954153_G_A		1.120	0.015	0.497	0.099	-2.124	0.701
XM_fecE_4508796_C_CCG		1.123	0.076	0.504	0.104	1.902	1.455
cvrALL_yajL_3		1.126	0.059	0.495	0.092	-3.915	4.598
cvrALL_ydeK_5		1.130	0.001	0.496	0.108	-1.252	2.231
XM_ydeK_1595330_A_AC		1.133	0.062	0.505	0.110	-3.268	0.936
cvrALL_slt_4		1.138	0.153	0.500	0.133	-12.104	7.905
XM_ydeK_1592148_G_GC		1.141	0.018	0.516	0.087	-1.313	0.935
cvrALL_yeJO_3		1.151	0.004	0.511	0.094	-7.135	0.505
XM_kgtP_2723967_G_GA		1.154	0.064	0.506	0.117	-1.137	0.695
XM_leuT_3980744_T_TC		1.156	0.101	0.502	0.082	1.029	2.691
XM_sstT_3239044_C_CG		1.158	0.125	0.505	0.126	-3.722	10.792
cvrALL_yfhM_3		1.159	0.041	0.505	0.095	5.437	3.366
cvrALL_hsdR_3		1.166	0.081	0.517	0.109	-5.652	4.905
XM_rnhA_235894_C_T		1.168	0.085	0.512	0.077	-1.805	2.092
cvrALL_ygbM_3		1.174	0.036	0.509	0.112	-0.760	1.075
XM_yihI_4049545_A_G		1.183	0.008	0.524	0.083	-0.925	0.073
XM_aceB_4214878_C_T		1.184	0.022	0.523	0.101	-2.336	2.685
XM_cytR_4122401_A_G		1.186	0.053	0.518	0.100	-5.291	3.337
XM_fixC_44802_C_T		1.187	0.083	0.510	0.118	-7.566	1.948
cvrALL_rbsA_3		1.188	0.113	0.538	0.040	-4.986	11.375
XM_ybaE_465768_C_T		1.189	0.083	0.515	0.081	0.121	3.856
XM_leuP_4604230_A_AG		1.191	0.052	0.526	0.072	-5.382	3.906
XM_frmR_379236_AG_A,AGG		1.194	0.008	0.518	0.100	-5.977	8.131
XM_entF_614896_A_G		1.197	0.069	0.526	0.096	-1.771	12.639
XM_pstA_3906598_G_GA		1.204	0.006	0.513	0.107	-0.975	0.464

XM_wza_2135543_A_G		1.214	0.020	0.522	0.095	1.098	2.474
cvrALL_ileS_3		1.268	0.195	0.528	0.055	-5.170	0.519
cvrALL_aceB_3		1.305	0.115	0.540	0.077	-9.457	7.816

Table S2. Assessing Causality of Alleles Identified via High Throughput Sequencing. To investigate the causality of the high-incidence mutations seen in the deep sequencing data set of dysfunctional *tolC* selection clones, we designed oligos to generate the exact mutations ("XM") seen with the highest incidence, and designed knockout oligos to cover all of the mutations seen in the coding regions mutated with the highest incidence ("cvrALL"). We performed a singleplex recombination with each oligo on EcNR2 (*tolC*$^+$), followed by a colE1 selection, to test the ability of each mutation in isolation to generate the dysfunctional phenotype. As controls, we recombined EcNR2 with 1, 2, & 5 μM *tolC*-r.null_revert oligo or water only. The former three constitute an important positive control that phenotypically defines "dysfunction" in this assay. The latter control (water) defines normal selection bleedthrough. Thus, mutations that contribute to dysfunction will lead to growth in the colE1 selection before the water control and likely after the *tolC*-r.null_mut controls. To distill growth data, we presented "Normalized Lagtime", "Normalized t @ V_{max}" (time where $d^2OD_{600}/dt^2 = 0$), and "% Change in V_{max}" (where V_{max}(EcNR2.ΔtolC) was used as reference). In the case of the normalized metrics, the average of *tolC*-r.null_revert wells was defined as 0, while the average of water control wells was defined as 1. Thus, normalized metrics for oligos bearing causal mutations fall between 0 and 1. All data are presented as the average & standard deviation of two independent replicates. All controls were performed for each replicate to account for assay-to-assay variability.

Table S3. Assessing Causality of Alleles Identified via High Throughput Sequencing.

Mutation ID	Normalized Lagtime ave	Normalized Lagtime st. dev.	Normalized t @ V_{max} ave	Normalized t @ V_{max} st. dev.
XM_*tolA*_775912_G_GA	-0.125	(n=1)	-0.083	(n=1)
tolC-r.null_mut	0.000	0.010	0.000	0.020
XM_Intergenic_752378_T_G	0.763	0.031	0.806	0.020
XM_recR_493693_C_T	0.827	0.020	0.847	0.020
XM_ogt_1398047_T_TC	0.892	0.010	0.875	0.059
cvrALL_treB_3	0.892	0.051	0.917	0.079
Water	1.000	(n=1)	1.000	(n=1)
gltA_mut	1.146	0.265	0.806	0.031
ybdG_mut	1.292	0.471	0.931	0.075

Table S3. Assessing Causality of Alleles Identified via High Throughput Sequencing. As in Table 1, except that recombinations to test these 4 mutations were done as a low-throughput singleplex recombination into EcNR2 before colE1 selection. To rule out the possibility that XM_intergenic_752378_T_G did not affect its proximal coding regions, ybdG and gltA, we also included oligos ybdG_mut and gltA_mut. As a positive control, we included a single replicate of XM_tolA_775912_G_GA. Assay controls included EcNR2 recombined with *tolC*-r.null_mut as well as with water, which defined Normalized Lagtimes of 0 and 1, respectively. The data are based on three biological replicates.

Table S4. Assessing Causality of Borderline Escape Mutations.

Strain ID		Normalized Lagtime ave	Normalized Lagtime st. dev.
EcNR2.Δ*tolC*		0.000	0.038
EcNR2.T752378G (intergenic)		0.924	0.043
EcNR2.T1398047TC (ogt)		0.935	0.041
EcNR2.C493693T (recR)		0.967	0.025
EcNR2.ΔtreB::zeoR		1.000	0.062
EcNR2 (*tolC*$^{+}$)		1.000	0.057

Table S4. Assessing Causality of Borderline Escape Mutations. Similar to Table 1 but here, monoclonal strains bearing the 4 mutations of interest delimit rows. As controls to define dysfunction, we included EcNR2.Δ*tolC* for which Normalized Lagtime ≡ 0. As controls to define normal bleedthrough, we included EcNR2, for which Normalized Lagtime ≡ 1. The data are based on three technical replicates.

Table S5. *btuB* Coding Mutations

btuB Mutation	Position	Type	Ref	Change	Mut. Result	Notes
1	4161989	DEL	G	-G	-1 Frameshift	Occurs before all of the 22 transmembrane domains
2	4162910	SNP	C	T	Premature Stop (Q437*)	Occurs within the 14th transmembrane domain
3	4163478	SNP	A	G	SNV (Y606C)	Occurs within the 22nd transmembrane domain (9 AA from C-terminus)

Table S5. *btuB* Coding Mutations. A list of btuB coding mutations from the high throughput sequencing data set.

Table S6. Oligonucleotides Used in this Study.

Set 27 TAA Oligos	ygfJ	c*c*ggacgactttattacagcgaaggaaaggtatactgaaatttaAaaaacgtagttaaacgattgcgttcaaatatttaatccttccg*g*c
	recJ	g*g*gattgtacccaatccacgctctttttttatagagaagatgacgTtaaattggccagatattgtcgatgataatttgcaggctgcggt*t*g
	argO	c*t*ctggaggcaagcttagcgcctctgttttatttttccatcagatagcgcTtaactgaacaaggcttgtgcatgagcaataccgtctc*t*c
	yggU	a*a*tccgcaacaaatcccgccagaaatcgcggcgttaattaattaAgtatcctatgcaaaaagttgtcctcgcaaccggcaatgtcggt*a*a
	mutY	g*t*ggagcgtttgttacagcagttacgcactggcgcgccggtttaAcgcgtgagtcgataaagaggatgatttatgagcagaacgattt*t*t
	glcC	g*c*caccatttgattcgctcggcggtgccgctggagatgaacctgagttaActggtattaaatctgcttttcatacaatcggtaacgct*t*g
	yghQ	a*c*tgagtcagccgagaagaatttccccgcttattcgcaccttccTtaaatcaggtcatacgcttcgagatacttaacgccaaacacca*g*c
	yghT	t*g*gttgatgcagaaaaagcgattacggattttatgaccgcgcgtggttatcactaAtcaaaaatggaaatgcccgatcgccaggaccg*g*g
	ygiZ	t*t*ctctgtctatgagagccgttaaaacgactctcatagattttaTtaatagcaaaatataaaccgtccccaaaaaagccaccaaccac*a*a
	yqiB	a*g*ggttaacaggctttccaaatggtgtccttaggtttcacgacgTtaataaaccggaatcgccatcgctccatgtgctaaacagtatc*g*c
Set 20 TAA Oligos	wcaL	g*g*aacgtgcgtcgggagagtttttaaatggcatgcgttctcctTtataaagcctgcagcaagctggcgagttctcgattgatcacct*g*c
	wcaC	t*t*ttgacgttaaccagcagataaatcagcggcagcaacaggtagTtacagattctgatagaagttgacatactcctccagcatctgtt*g*t
	asmA	c*t*ttacgcgtcttatcaggcctacaggactggtcacctggtaacTtacatcttctccagcaacttcttcacatctttgccattacggg*a*a
	baeR	c*c*gcgccgtttatggcgtcggttaccgctgggaagccgacgcctgccgcatcgtttaAttttagcgacattattttgttagccggaga*t*g
	gatR_1	t*g*ccggatgcggtgtaaacaccttatccggcatacagaacaataTtacaccgtaattaattttacccgcgtcttctgcaatgccagtt*t*a
	tra5_4	g*g*cggcctcagtccggaacaatttgaaaacaagaacctcgcttaAgcctgtgtccatattacgtgggtaggatcagtcatcccttcgt*g*g
	yegV	g*c*gccaacgcgcgaggaactactcctcgcacacaaaaacgtataAatcgctgcgacagtggctaatgctgtactcaataggccgttgc*t*g
	yegW	g*c*gtcgtgggttgtcgggcatcggggcggtgattgtgcgccaacgcgcgaggaaTtactcctcgcacacaaaaacgtatagatcgctg*c*g
	yehQ	a*a*cggctggcgctatcaggaacagagtgctattggtcaggcataAttgctggcgaatgacctcaccgcggtgggtttttatcggctgg*c*a
	yohC	c*g*tcaaaggagaatcgtgaggaaatgctgcattgctgacatttacgccagcaatgcaacgtcaaagaactttTtagaacaaacgataa*c*c
Set 3 masPCR	recJ_wt-f	TCATCGACAATATCTGGCCAATTTAG
	argO_wt-f	TGCACAAGCCTTGTTCAGTTAG
	yggU_wt-f	CAGAAATCGCGGCGTTAATTAATTAG
	mutY_wt-f	GGCGCGCCGGTTTAG
	glcC_wt-f	GCTGGAGATGAACCTGAGTTAG
	yghQ_wt-f	CTCGAAGCGTATGACCTGATTTAG
	yghT_wt-f	CGCGCGTGGTTATCACTAG
	ygiZ_wt-f	TGGGGACGGTTTATATTTTGCTATTAG
	yqiB_wt-f	CGATGGCGATTCCGGTTTATTAG
	ygfJ_mut-f	AGCGAAGGAAAGGTATACTGAAATTTAA
	recJ_mut-f	TCATCGACAATATCTGGCCAATTTAA
	argO_mut-f	TGCACAAGCCTTGTTCAGTTAA
	yggU_mut-f	CAGAAATCGCGGCGTTAATTAATTAA
	mutY_mut-f	GGCGCGCCGGTTTAA

	glcC_mut-f	GCTGGAGATGAACCTGAGTTAA
	yghQ_mut-f	CTCGAAGCGTATGACCTGATTTAA
	yghT_mut-f	CGCGCGTGGTTATCACTAA
	ygiZ_mut-f	TGGGGACGGTTTATATTTTGCTATTAA
	yqiB_mut-f	CGATGGCGATTCCGGTTTATTAA
	ygfJ_rev	GATGAACTGTTGCATCGGCG
	recJ_rev	CTGTACGCAGCCAGCC
	argO_rev	AATCGCTGCCTTACGCG
	yggU_rev	TAACCAAAGCCACCAGTGC
	mutY_rev	CGCGAGATATTTTTTCATCATTCCG
	glcC_rev	GGGCAAAATTGCTGTGGC
	yghQ_rev	ACCAACTGGCGATGTTATTCAC
	yghT_rev	GACGATGGTGGTGGACGG
	ygiZ_rev	ATCGCCAAATTGCATGGCA
	yqiB_rev	AAAATCCTGACTCTGGCCTCA
tolC Inactivation/Reversion Oligos	tolC-r.null.mut	A*G*CAAGCACGCCTTAGTAACCCGGAATTGCGTAAGTCTGCCGCtaaATCGTGATGCTGCCTTTGAAAAAATTAATGAAGCGCGCAGTCCA
	tolC-r.null.revert	A*G*CAAGCACGCCTTAGTAACCCGGAATTGCGTAAGTCTGCCGCCGATCGTGATGCTGCCTTTGAAAAAATTAATGAAGCGCGCAGTCCA
	tolC.null.mut	T*G*GACTGCGCGCTTCATTAATTTTTTCAAAGGCAGCATCACGATttaGCGGCAGACTTACGCAATTCCGGGTTACTAAGGCGTGCTTGCT
	tolC.null.revert	T*G*GACTGCGCGCTTCATTAATTTTTTCAAAGGCAGCATCACGATCGGCGGCAGACTTACGCAATTCCGGGTTACTAAGGCGTGCTTGCT
Endogenous tolC Deletion Oligo	tolC.90.del	gaatttcagcgacgtttgactgccgtttgagcagtcatgtgttaaagcttcggccccgtctgaacgtaaggcaacgtaaagatacgggttat
Endogenous tolC Sequencing Primers	Rx-P19	GTTTCTCGTGCAATAATTTCTACATC
	Rx-P20	CGTATGGATTTTGTCCGTTTCA
tolC Cassette Primers	tolC_wt-f	ATGTGAATTTCAGCGACGTTTG
	tolC_wt-r	GCAGATAACCCGTATCTTTACGT
	2223750.T.20.21r-f	TGC CAG TAA GCA TAC ACC CTG TGA ATC ATT CAC GCT GAA AAA GGA CGT TCT TGA GGC ACA TTA ACG CC
	2223749.T.20.21r-r	GTC AGC TTG TGA TTA TTT TTT GTT CGC CCA TGT AAT TTT CAC TGT CTG ATT CTA GGG CGG CGG ATT
tolQRA Duplication Cassette/Sequencing Primers	Ec1984562::tolQRA-1-f	GGGAAAAGACATAAGGGAAAGCCAATTTGTCAGACAAATTGTCGA***TGGGCCGCTCCAGGCCCATAAATTTTTACGCTCCCTTAACTTGCC***
	Ec1984562::tolQRA-1-r	**TATTGACTGGTTAAAAAGAAGACATCCCGCATGGGTACCAAAGACTTACGGTTTGAAGTCCAATGGCGCGTTTTTGAACACTTCATATAC**
	Ec1984562.seq-f	*CGCAGAATTACAGTGAGAACGTG*
	Ec1984562.seq-r	AGGTAATGCGGCC**TATTGACT**
	Ec1984562::tolCins-1-f	AAAAGACATAAGGGAAAGCCAATTTGTCAGACAAATTGTCGA*TTGAGGCACATTAACGCC*
	Ec1984562::tolCins-1-r	**ATTGACTGGTTAAAAAGAAGACATCCCGCATGGGTACCAAAGAC***TCTAGGGCGGCGGATT*
	Ec1255700::tolQRA.1-f	TCTTGCCAGCATATTGGAGCGTGATCAATTTTGATCAGCTGTGAA***TGGGCCGCTCCAGGCCCATAAATTTTTACGCTCCCTTAACTTGCC***

	Ec1255700::tolQRA.1-r	*CAGTGATATAACGTAAGTTTTTGTATCACTACACATCAGCCCCCT***TTACGGTTTGAAGTCCAATGGCGCGTTTTTGAACACTTCATATAC**
	Ec1255700::tolCins-1-f	TGCCAGCATATTGGAGCGTGATCAATTTTGATCAGCTGTGAA*TTGAGGCACATTAACGCC*
	Ec1255700::tolCins-1-r	*TGATATAACGTAAGTTTTTGTATCACTACACATCAGCCCCCTTCTAGGGCGGCGGATT*
	Ec1255500.seq-f	catttttgcattactaataagaaaaagcaaa
	Ec1255850.seq-r	GTCCTAATCATTCTTGTAACATCCTAC
	tolQRA.tolCins-f	**GTATATGAAGTGTTCAAAAACGCGCCATTGGACTTCAAACCGTAA**TTGAGGCACATTAACGCCCT
	tolQRA.tolCins.1255700-r	*CATTAGCAGTGATATAACGTAAGTTTTTGTATCACTACACATCAGCCCCCT*TCTAGGGCGGCGGATTTGTC
	tolQRA.btuBins-1-f	**GTATATGAAGTGTTCAAAAACGCGCCATTGGACTTCAAACCGTAAGCCGGTCCTGTGAGTTAATAGG**
	tolQRA.btuBins.1255700-r	*CATTAGCAGTGATATAACGTAAGTTTTTGTATCACTACACATCAGCCCCCT***TCAGAAGGTGTAGCTGCCAG**
Nuclease KO Reversion Oligos	xonA_mmKO.rev	A*A*T*A*ACGGATTTAACCTAatgATGAATGACGGTAAGCAACAATCTACCTTTTTGTTTCACGATTACGAAACCTTTGGCACGCACCC*C*G*C*G
	recJ_mmKO.rev	G*G*G*A*GGCAATTCAGCGGGCAAGTCTGCCGTTTCATCGACTTCACGGCGACGAAGTTGTATCTGTTGTTTCACGCGAATTATTTACC*G*C*T*G
	xseA_mmKO.rev	G*A*A*T*TTGATCTCGCTCACatgTTACCTTCTCAATCCCCTGCAATTTTTACCGTTAGTCGCCTGAATCAAACGGTTCGTCTGCTGCT*T*G*A*G
	exoX_mmKO.rev	T*T*C*G*GCCTGGAGCATGCCatgTTGCGCATTATCGATACAGAAACCTGCGGTTTGCAGGGAGGGATCGTTGAGATTGCCTCTGTTGA*T*G*T*C
Nuclease KO/Primase Mutation Allele-specific Primers	exoX.KO*-wt-f	GCGCATTATCGATACAGAAACCt
	recJ.KO*-wt-f	CAACAGATACAACTTCGTCGCc
	xonA.KO*-wt-f	GAATGACGGTAAGCAACAATCTacc
	xseA.KO*-wt-f	CTTCTCAATCCCCTGCAATTtttacc
	Lexo_WT-f	GGCAGCATGACACCGGA
	dnaG_Q576A_wt-f	TGGAGCTCTGGACATTAAACCA
	exoX.KO*-mut-f	gcgcattatcgatacagaaacTGA
	recJ.KO*-mut-f	caacagatacaacttcgtcgTGA
	xonA.KO*-mut-f	gaatgacggtaagcaacaatcTGA
	xseA.KO*-mut-f	cttctcaatcccctgcaatTGA
	Lexo_MUT-f	TGGCAGCATGACACCGTAA
	dnaG_Q576A_mut-f	GGAGCTCTGGACATTAAACGC
	exoX.KO*-r	GACCATGGCTTCGGTGATG
	recJ.KO*-r	GGCCTGATCGACCACTTCC
	xonA.KO*-r	GAAATGTCTCCTGCCAAATCCAC
	xseA.KO*-r	GGTACGCTTAAGTTGATTTTCCAGC
	Lexo-r	CAAGGCCGTTGCCGTC
	dnaG_seq-r	GCTCCATAAGACGGTATCCACA
Prophage Deletion Cassette/Sequencing Primers	dlambda::zeoR-f	GGCTATGAAATAGAAAAATGAATCCGTTGAAGCCTGCTTTTTTATACTAA**GGTGTTGACAATTAATCATCGGC**
	dlambda::zeo	GCCGCGTTGATTTTCTCCTGCCAGCTCATAATGCTGCCGCGTTGTAATAT**AG**

	R-r	**CTTGCAAATTAAAGCCTTCG**
	Lambda.Seq-f	CAAAAGCCAATGCCAGC
	Lambda.Seq-r	TTCAGATACTGGCGATCATC
Oligos to Assessing Causality of Escape Mutations	XM_tolA_776493_C_CG	C*A*CTCCCGGCAGGCGAAGCATTGTTCCCTTTCGCCCCTCCCCCCCGTTTTCGGTGCATTCTTACCAGAGCTTAGCTCACCGAAAATAT*C*A
	XM_wza_2135543_A_G	T*T*CACACCCTCTCAGCATGCAGTCGTTGATGAGAAAGGGTTATTgCGGAAATTAACTTCCGAATATAAGGTGACATTATGGTAATTGA*A*T
	XM_frmR_379236_AG_A,AGG	C*C*GCTATCCGGGGCGGCCTTCCCTGCCGATTAGCCCCCCCCCCCTTTTCCTCTTTGTTTTCCGACCACATTCACCGGATAAATTTTAT*T*C
	XM_ydiM_1769931_G_A	G*C*TCCACTCATTCGTAATACCGTTCGCCCAACAACATTACTGATaCTGTACACCTTTATCTCATTTATTGCTCTGTTTACCGTCTGCC*T*G
	XM_Rep321e_4294083_C_T	T*G*TCTGGCGTAGGCCTGATAAGACGCGGCAAGCGTCGCATCAGGCATTGATGTCGGATGCGGCGTAAACGCCTTATCCGACCTACAAA*A*T
	XM_rnhA_235894_C_T	T*A*CGGCGCTATTTTACGCTATCGCGGACGCGAGAAAACCTTTAGCACTGGCTACACCCGCACCACCAACAACCGTATGGAGTTGATGG*C*C
	XM_tolA_776256_AGG_A	T*G*TCGGCTGCAGCTTTCTCTGCTGCCGCTTTCTTATCAGCTGCAGTTTTCAGCAGCCGCTTTCTTCTCAGCTTCTGCTTTGGCTTTTT*C*A
	XM_hyfR_2610011_G_A	A*C*AATTGAAGCGGTAAACGGAATGCTCGCGGAGCGGTTAGCACAaAAACACGGCAAGGCGTCTTTATTACGCGCCTTCATCCCGCTGC*C*G
	XM_yjgF_4468858_T_A	G*C*AGCTATCGGTCCTTACGTACAGGGCGTTGATCTGGGCAATATGTTCATCACCTCCGGTCAGATCCCGGTAAATCCGAAAACGGGCG*A*A
	XM_ybaE_465768_C_T	G*A*TTACTGGCTTGCGCACCGGCTGGCGAGCTATTGCAGCCATCTGACGCATCCGCAATTCCCACTGATCGGCACGGGTCCTTTTCGCT*T*A
	XM_tolQ_774584_A_AG	T*G*TAAAAGATTTGTTCCGAACCAGTCAGATTATCCCGTTTCCCCCTGGCTCTCTTGATAGAGGCGAGAGAGTTCGATTCCAGACCAGA*A*T
	XM_tolA_775912_G_GA	T*G*CTTTAACTCGGCCTGTTTTGCGGCTTCTTCAGCCTGCTTTTTTCTGCTCCTGAGCCGCTAACCGCTCTTTCTCAAGTTGCTTCAGG*C*G
	XM_fixC_44802_C_T	C*C*GCCCATCAGGCCATCGGTGGGTGATCCCGCAAACAGGCAAGCCACCCCCTGATTACCCTGCAACTGAAAACGGTCTTCAATAACCG*A*C
	XM_entF_614896_A_G	G*C*CAGGGTCAAAAAGACCGAGCGCGGTAGTGCCACCGCCACGCTGCCCCCTGGTTTAACGCCGCGCTCACGCAGCAGATTCGCCAGCG*C*C
	XM_tolQ_774693_A_AG	T*C*AAGTTCACGGTTCATGGAGATACGCATAGCACGCGACGCCCCCTTCCACTACGGCTTCCGGCGCATGGCTATTGGCACGATGCAGG*C*G
	XM_tolR_775139_A_G	A*T*GGGCGCTGTCGCCATAAAGATCAGCAACAGCACCAGCAGTACGCCCAGCAACGGTACAATGTTGATTTCGGACTTGAGATCGCGAC*G*A
	XM_tolR_775339_C_T	T*T*TTCGGGTTGGCCTTGAAACGGCTGGACACTTCCGCCACCACCTACTCTGGTGGTAAACGCTCCAGGCGATCTTTCTCAACCACCAC*G*G
	XM_tolA_776113_GA_G	T*C*CGCTTTCTTCTTCAGTGCCGCAGCGGCTGCCTCGGCTTTTTTTCTGCGCTTCGGCTGCGGCTTTGGCGGCTTCTGCTTCTGCTTTT*T*T
	XM_tolA_776380_CA_CAA,C	C*A*GCGGCTTTTTCAGCTGCAGCTTTTGCCGCTGCCGCTTTTTGTTGTCTGCGGCGGCTTTTTCTGCCGCTGCTTTCTTATCAGCTGCT*G*C
	XM_iraM_1211303_A_AC	A*T*ATAAGCCATCATTTTAATTAATTTATTTTTTTGAGGGGGGGGGTAATATACTCATATGCAAAATCAAGAAATAAACATCCTAATGA*A*C
	XM_tynA_1449056_G_A	A*G*TGATGAAAGACAATAAAGCCTGGGTTTCTGACACCTTTATTAATGATGTTTTCCAGTCCGGGCTGGATCAAACCTTTCAGGTAGAA*A*A
	XM_ydeK_1592148_G_GC	A*A*TGTCACCACTGCAGGTGATATGAACGTTATGCCTGGGGGGGGGCACTGCGTGTCGCTAAAACCACTATCGGCGGCAACCTGGAGAA*T*G
	XM_ydeK_1595330_A_AC	T*T*GGTAATCAAGGAACTGGGGAGGCGACTATTCGCGAGGGGGGGGTTGGTTACGGCTGAAAATACGATTATCGGTGGCAATGCCACCG*G*T
	XM_insE5_2168234_T_C	C*G*GTCTGATCTTACCCAGCAATAGTGGACACGCGGCTAAGTGAGcAAACTCTCAGTCAGAGGTGACTCACATGACAAAAACAGTATCA*A*C
	XM_kgtP_2723967_G_GA	A*A*ATTTCTGGTAAGGAGGACACGTATGGAAGTGGGCAAGTTGGGGaAAGCCGTATCCGTTGCTGAATCTGGCATATGTGGGAGTATAA*G*A
	XM_ilvA_3954153_G_A	C*G*CACATCTTCGAATAAATCCTTCATCGCCGCACAGATCGCATCGTTATCGACGGTGATGATGTCGTCGAGATACTCCTGGCATAAAC*G*G
	XM_ftsZ_105469_A_T	A*G*CGCCCAGTCCTTTGGTGATACCGCTACCGATTTGAATCGTCTGACCAACCGCTGTTTTACGCAGCGCTTGTGCATCGGTATTTACC*G*C
	XM_recR_493693_C_T	C*G*ATCGCGCTGAAGCAGCGTGAACGCCATACGCTGCGCCGACTTCAGGCCAACGCCCGGCAGACAGCGCAGTGCTTCCATAAGCTGTG*T*T
	XM_Intergenic_752378_T_G	C*G*ATATCAAGCGTTAATGGTTGATTGCTAAGTTGTAAATATTTTACCCCGCCGTTCATATGGCGGGTTGATTTTTATATGCCTAAACA*C*A
	XM_tolQ_774558_A_ACT	G*A*TTATCCCGTTTCCCCTGGCTCTCTTGATAGAGGCGAGAGAGAGTTCGATTCCAGACCAGAATTTATCTTCAAACGCTTCGGCTTCG*C*G

	XM_tolA_775823_C_T	G*T*TCCTGTTCAGCCGCTTGTTTCTCACGCAGTTCTTCAGCAGCCTACTGTTCCTTCATCTTGCGCTGTTCATCAGAACGCTTCGCGCT*T*G
	XM_tolA_776642_GGCAA_G	A*G*TAACATACCATCGGGTGCCAGTTTTATGCGCAGCGTACAGGTTCTGCATAGGACGATGCGTCATAGAACTTACTTTCGATAGCAGA*T*T
	XM_ycgB_1235495_A_AG	T*G*GAGTTTTTGCACAGCCACACCAATGTGGTCTTCCAGCCCCCCCTATAACAGCCCGTGGTACAGCGGCATCAACCCGTATGCCCTCG*G*G
	XM_ynfE_1658003_G_A	T*A*CAAAGCATTTCGTGACGACCCCGAGGCAAATCCACTTAAAACaCCTTCCGGTAAGATTGAAATTTATTCCAGCAGGCTGGCGGAAA*T*T
	XM_yfiH_2732557_T_TC	C*T*GCACTTGCTTTAGCGTCTACTGCCATAAACGCCTCGCGAACCTcCCCCCCCCACTTCGAACGCGCGTGGACCAATTGCCGGCCCTA*A*C
	XM_yihI_4049545_A_G	A*A*TGGAATAGGGGTTTTACTGCCAATACGTGGATCTTTTGGTGCGCTCTGGCCTTTGCTGCCTGACGTGGTGTTTCCGCCCGCTGCGC*G*G
	XM_cytR_4122401_A_G	A*T*GAAAGACGTTGCCCTCAAGGCAAAAGTCTCTACAGCGACCGTCCCCCGAGCATTAATGAATCCCGATAAAGTCTCCCAGGCCACCC*G*T
	XM_aceB_4214878_C_T	T*C*ACCGGTTTGCCATTGCTCAACGTTTTTTGATGATGGATCCACTACCAGATCGAGGTACGGGAAATTTCAGCCGTCGCCGCATCTTC*C*A
	XM_leuP_4604230_A_AG	A*G*GTGTTAGTGTTCTTACGGACGTGGGGGTTCAAGTCCCCCCCCCTCGCACCAAACGAGGCGATATCAAAAAAAGTAAGATGACTGTG*C*G
	XM_eaeH_313960_T_TC	C*T*TTATCGACATTCAGTTTGACGCGCGCAGTACCATATTTCCCGGAGCCACTCCTGTATTTCCTGGTTAGCTTTAGCTGTGGCCATTC*C*G
	XM_fepE_617633_C_CG	G*C*AGGCAAACGCAAAAACGACCGCCATGACCGTTTTTTTGGCCCCGCCATAAAACTGAGATTAGATTAAGTAAATCAATTTCATTATT*A*C
	XM_elfC_1000951_C_CG	C*T*TCGCCCCAGCGCACGCTTATTGTTCCCTTCGCCGGTAAACCCCGCCATATAGAGCGAGCCATCATCATCAATTATGCCGCTATTAG*C*C
	XM_ogt_1398047_T_TC	G*T*AATCTTAGCATTATTGATACGCTTCCCACTGCTACGGGGGGGGACGCCATTTCAGCGCGAAGTCTGGAAAACACTACGCACTATCC*C*C
	XM_ydeK_1592317_T_TC	C*G*ATTCCGCGGCGTATCAGGGTACGACGGATATCGTGGGGGGGGGAAATTGCTTTCGGTTCCGACTCTGCCATTAATATGGCAAGTCA*A*C
	XM_fumC_1683605_GC_G	A*T*CACCATTGGACGGAAGACGTTCAGTTCAAAGTTACCGGAAGCgCCCCCATGTTGATCGCCACGTCGTTCCCCATCACCTGACAGCA*G*A
	XM_yehA_2186104_G_GC	G*C*ACTGGATAATGTCGCAACACCATTAATCATCACTGAACCGCCGcCCCCCCGCAGAAACATTCACTTTTGCATTACTGGCCGCAGGT*G*T
	XM_hyfB_2600077_G_GC	G*A*ATTACGCAATTACATGCTGGTGTAACTTTAGTCGCCCGTTTTGcCCCCCCCTTTTGAATTTGCCGATTTAACCCTGCGAATGGATA*G*C
	XM_sstT_3239044_C_CG	G*A*GCGTGGTGGCTTCTCTGTGTGCCTGTGGCGCATCCGGCGTGGCgGGGGGGGTCTCTGCTGCTGATCCCACTGGCCTGTAATATGTT*C*G
	XM_pstA_3906598_G_GA	C*G*GTCGGTCCCCTCGCCCCTCTGGGGAGAGGGTTAGGGTGAGGGGaAAAACCGTGTCAGCAATATCAACCGTGTTTATTCTTCGCAAA*A*A
	XM_yifK_3979054_G_GC	T*C*ATCGCCCTGGGGGGCACCATTGGCGTCGGCCTGTTTATGGGGGcCCGCCAGTACCCTGAAATGGGCCGGGCCATCCGTATTGTTGG*C*C
	XM_leuT_3980744_T_TC	G*C*TAGCTTCAGGTGTTAGTGTCCTTACGGACGTGGGGGTTCAAGTcCCCCCCCCTCGCACCACGACTTTAAAGAATTGAACTAAAAAT*T*C
	XM_polA_4045942_A_AC	G*C*TGATGTCGAAGCGGGCAAATGGTTACAGGCCAAAGGGGCAAAAcCCAGCCGCGAAGCCACAGGAAACCAGTGTTGCAGACGAAGCA*C*C
	XM_fecE_4508796_C_CCG	T*G*CAATCTACCTCATTAGGCACATCGGCCTGCCAGATACCGGCTCcgGGGGTGTATTTCCGCTTCCACGCTGAATACTGTTCTCAGCA*A*T
	XM_leuV_4604154_A_AG	A*A*ATCAAGGAAGAAACAAGAAAGGAAGTAAAGATAATTGGTGCGAgGGGGGGGGACTTGAACCCCCACGTCCGTAAGGACACTAACAC*C*T
	cvrALL_tolA_23	G*C*CCAAGCGGAAAATGCCGTGACGGAACACGCCGTCAGCAGCGCTAAGCTTTTTTAATCATTGTAAAGCATCCACAATAGAAGAAGGA*T*G
	cvrALL_tolQ_14	C*A*TGATAAGTTTAACCAGAAGGCTAGCCTTCAGGAACAAATCACAGGATATTCATGTCAGTCACTGCTTAAACTCCGCGACAATAGAC*T*T
	cvrALL_tolR_8	C*A*GCAGTACGTCCAGCAACGGTACAATGTTGATTTCGGACTTAGAGATCGCGACGACCTCGTCCACGCGCTCTGGCCATGGCTTACCC*C*T
	cvrALL_lhr_5	A*T*ATTCACGGGGAATAGCGTTAAatgGCAGATAATCCAGACCCTTaaCATCGCTCCTGCCGGACGTGTTTTCACCGGCGACCCGCGACT*G*G
	cvrALL_uvrB_5	C*G*AATCGCCTCTGGCTGATCGCCAGAAGGTTTAAAAGCGGAATTACAGTTTGAACGGTTTACTCATGAGTCGCTACCTGAAGGAGTTG*G*G
	cvrALL_ydeK_5	C*G*AGTTAATTCCGAGCAGGCCTGAAATACCTGTAGAGTGCAATTACCATATCACGCGATAGATTCTATTCATATAAACGCTCCATATA*C*A
	cvrALL_slt_4	G*C*TGCTGACCGTCAGCAGACAGACACCGGCAGCCAACAGCCGCCTAGGTAACTTGTTTGGCTTTTTCCACAAGCGCATCCTCTAAGTG*T*A
	cvrALL_aceB_3	T*T*TGCTTCTCCTGCTCGCCATACGGCCTTGTGAAAGCCAGTTCAATCGGTTGTTGTTGCCTGTTCAGTCATCGTGCAGCTCCTCGTCA*T*G
	cvrALL_btuB_3	T*C*CTGTGCCCAAGCGGAAAATGCCGTGACGGAACACGCCGTCAGTGCAGCGAAGCTTTTTTAATCATTGTAAAGCATCCACAATAGAA*G*A

	cvrALL_hsdR_3	T*T*TATTCATCATTGTTATTAATCCATTGCTGTGCGGGCCTGTCCTAAATATTTAAGGCCCATAACATCTCATCTTAGCTTTCTGTACC*T*T
	cvrALL_ileS_3	C*A*CGCATCGGGAACCCTGTTTCCGGCAAATTCAGGGTTGATTTATTAGTCACTCATCAGATTCTCGGTTCCGTATTTCGGTTTGATTA*C*A
	cvrALL_malZ_3	G*T*TGATCTTTGCTTTGTTTAACAAATGGGGGCACCGGCAGGTGCTCATGCATTTAACATCATAATTCCCCTGTGATGAAAATCTGATC*A*C
	cvrALL_rbsA_3	G*C*TGGCGTGACGTTCTGAGGCCGTCatgGAAGCATTACTTCAGCTaTAAAGGCATCGATAAAGCCTTCCCGGGCGTAAAAGCCCTCTC*G*G
	cvrALL_recE_3	G*C*CCACAGGACGACGTCAGGTTCACCGGATGATTTTTTCGCTTTACCGTAACAGGAAGAGTGGTTTTGTGCTCATTGTTTTTTAACCT*C*A
	cvrALL_tolC_3	T*C*ACCACAAGGAATGCAAatgAAGAAATTGCTCCCCATTCTTATCaaGGCCTGAGCCTTTCTGGGTTCAGTTCGTTGAGCCAGGCCGA*G*A
	cvrALL_treB_3	A*T*ATTGCCGCGCCCGCCGACCAGTTCAATCAACCGATCGATATCACGTTTGGTTTATTTTGCTCATCATAAAGCCCCATGGCAGATGA*C*A
	cvrALL_yajL_3	C*G*ATAGTGGTGACGGCTTCAGTCTCTTCACTACCAGGGGCGAGGTCAAACCAGTGCCGATGCGCTCATATTCACTCTCCTTTCTTTTT*A*C
	cvrALL_yeJO_3	A*C*CTTCCTTATATTCACCCATAGCAttgGCATCAACTGTTGAGTAaTGGTGAGACAGTTGATGGTGTTGTCCTGGAAAAAGATATCCA*G*C
	cvrALL_yfhM_3	A*T*TGTGGCAGGGATGAAAAatgAAAAAGTTACGCGTAGCCGCCTGaCATGCTAATGCTGGCGCTGGCAGGGTGCGACAACAACGATAA*C*G
	cvrALL_ygbM_3	G*A*AATTGCGGAATTAAGGAGTTAATGCAatgCCTCGTTTTGCAGCaTAATTTATCCATGATGTTCACCGAAGTGCCTTTTATTGAACG*C*T
	tolC_3176270_A_G	G*C*AAGCACGCCTTAGTAACCCGGAATTGCGTAAGTCTGCCGCCGGTCGTGATGCTGCCTTTGAAAAAATTAATGAAGCGCGCAGTCCA*T*T
	tolC_3176564_A_G	C*C*TCAACACCGCGACCGCTTATTTCAACGTGTTGAATGCTATTGGCGTTCTTTCCTATACACAGGCACAAAAAGAAGCGATCTACCGT*C*A
	tolC_3176582_A_G	T*T*ATTTCAACGTGTTGAATGCTATTGACGTTCTTTCCTATACACGGGCACAAAAAGAAGCGATCTACCGTCAATTAGATCAAACCACC*C*A
	tolC_3176753_A_G	G*A*CCGCACGTAATAACCTTGATAACGCGGTAGAGCAGCTGCGCCGGATCACCGGTAACTACTATCCGGAACTGGCTGCGCTGAATGTC*G*A
	tolC_3176813_A_G	C*T*ATCCGGAACTGGCTGCGCTGAATGTCGAAAACTTTAAAACCGGCAAACCACAGCCGGTTAACGCGCTGCTGAAAGAAGCCGAAAAA*C*G
	tolC_3177340_T_C	G*T*ACGCGTACCATTGTTGATGTGTTGGATGCGACCACCACGTTGCACAACGCCAAGCAAGAGCTGGCGAATGCGCGTTATAACTACCT*G*A
	tolC_3176840_T_C	C*G*AAAACTTTAAAACCGACAAACCACAGCCGGTTAACGCGCTGCCGAAAGAAGCCGAAAAACGCAACCTGTCGCTGTTACAGGCACGC*T*T
	dtreB::zeoR-f	GCGCAATATATTCTGCAGCCAACCAAAAATGTCATCTGCCATGGGGCTTTGGTGTTGACAATTAATCATCGGC
	dtreB::zeoR-r	TTACGTTATTCCTGCGAATGCGAGGGGGCGCAATTGCGCCCCGAAGAAAAAGCTTGCAAATTAAAGCCTTCG
tolC_ssrA Cassette Primers	tolC_ssrA-Nr	tcaagctgctaaagcgtagttttcgtcgtttgcgacgttacggaaagggttatgaccg
	tolC_ssrA-Cf	ACgtcgcaaacgacgaaaactacgctttagcagctTGAAAGCTTGATATCGAATTCCTGC
	tolC_ssrA_weak-Nr	tcaagaagcgtcagcgtagttttcgtcgttagcagcgttacggaaagggttatgaccg
	tolC_ssrA_weak-Cf	ACgctgctaacgacgaaaactacgctgacgcttctTGAAAGCTTGATATCGAATTCCTGC
	tolC_ssrA_int1-Cf	ACgtcgcaaacgacgaaaactacgctttagcatctTGAAAGCTTGATATCGAATTCCTGC
	tolC_ssrA_int1-Nr	GCAGGAATTCGATATCAAGCTTTCAAGATGCTAAAGCGTAGTTTTCGTCGTTTGCGACGT
	tolC_ssrA_int2-Cf	ACgtcgcaaacgacgaaaactacgctgacgcagctTGAAAGCTTGATATCGAATTCCTGC
	tolC_ssrA_int2-Nr	GCAGGAATTCGATATCAAGCTTTCAAGCTGCGTCAGCGTAGTTTTCGTCGTTTGCGACGT
tolC_ssrA Promoter MAGE Oligos	TssrAopt_promoterMAX*	GACAAATTGTCGATTGAGGCACATTAACGCCttGaCAgctagctcagtcctaggtaTaatgctagcCGCAATAATTTTACAGTTTGATCG
	TssrAopt_promoter*	GACAAATTGTCGATTGAGGCACATTAACGCCttKaYRgctagctcagtcctaggtaYaatgctagcCGCAATAATTTTACAGTTTGATCG
	TssrAopt_RBS*	TAATTTTACAGTTTGATCGCGCTAAATACTGCTTCACCACARGRRRTGCAAATGAAGAAATTGCTCCCCATTCTTATCGGCCTGAGCCTT

	TssrA33_97111opt	G*G*CTGCAGGAATTCGATATCAAGCTTTCAagctgctaaagcgtagttttcgtcgtttgcgacGTTACGGAAAGGGTTATGACCGTTACTG
	TssrA24_97111opt	C*A*GGAATTCGATATCAAGCTTTCAAGCTGCTAAagcgtagttttcgtcgtttgcgacGTTACGGAAAGGGTTATGACCGTTACTGGTGGT
2-step mutS Replacement Cassette Primers	mutS_wt-f	ACCCCatgAGTGCAATAGA
	N-mutS_wt-r	CAAACTCGCCCATCTTCTC
	tolC+N-mutS_wt-f	TGTCGATAGTGCACCGGTACAGGCGCTACGTGAGAAGATGGGCGAGTTTGTTGAGGCACATTAACGCC
	mutS_wt+tolC-r	gtcagttgtcgttaatattcccgatagcaaaagactatcgggaattgttaTCTAGGGCGGCGGATT
	tdk+N-mutS_wt-f	TGTCGATAGTGCACCGGTACAGGCGCTACGTGAGAAGATGGGCGAGTTTGCTGTTGAGCTGAAAAACTGAC
	tdk+mutS_wt-r	gtcagttgtcgttaatattcccgatagcaaaagactatcgggaattgttaCTGCCGAGAAGGGTATATAGC
	C-mutS-f	GTGCGCAGTTAGAAACTGTC
	C-mutS-r	gtcagttgtcgttaatattcccgatag
mutS Sequencing Primers	mutS.seq-f	GATACAATTTTGCGTACTTGCTTC
	mutS.seq-r	GTGTAGATGGCATGGTTTTAC
Barcoded Illumina Adaptors for Multiplexed Re-sequencing	p5	AATGATACGGCGACCACCGAGATCTACACACTCTTTCCCTACACGACGCTCTTCCGATCT
	P7_A1	CAAGCAGAAGACGGCATACGAGATcaggttGTGACTGGAGTTCAGACGTGTGCTCTTCCGATCT
	P7_A2	CAAGCAGAAGACGGCATACGAGATtcacaaGTGACTGGAGTTCAGACGTGTGCTCTTCCGATCT
	P7_A3	CAAGCAGAAGACGGCATACGAGATacatcaGTGACTGGAGTTCAGACGTGTGCTCTTCCGATCT
	P7_A4	CAAGCAGAAGACGGCATACGAGATagcgcaGTGACTGGAGTTCAGACGTGTGCTCTTCCGATCT
	P7_A5	CAAGCAGAAGACGGCATACGAGATcatcaaGTGACTGGAGTTCAGACGTGTGCTCTTCCGATCT
	P7_A6	CAAGCAGAAGACGGCATACGAGATgctattGTGACTGGAGTTCAGACGTGTGCTCTTCCGATCT
	P7_A7	CAAGCAGAAGACGGCATACGAGATtagatcGTGACTGGAGTTCAGACGTGTGCTCTTCCGATCT
	P7_A8	CAAGCAGAAGACGGCATACGAGATcatgacGTGACTGGAGTTCAGACGTGTGCTCTTCCGATCT
	P7_A9	CAAGCAGAAGACGGCATACGAGATgaatcgGTGACTGGAGTTCAGACGTGTGCTCTTCCGATCT
	P7_A10	CAAGCAGAAGACGGCATACGAGATtcttctGTGACTGGAGTTCAGACGTGTGCTCTTCCGATCT
	P7_A11	CAAGCAGAAGACGGCATACGAGATattccgGTGACTGGAGTTCAGACGTGTGCTCTTCCGATCT
	P7_A12	CAAGCAGAAGACGGCATACGAGATggaattGTGACTGGAGTTCAGACGTGTGCTCTTCCGATCT
	P7_B1	CAAGCAGAAGACGGCATACGAGATacggtgGTGACTGGAGTTCAGACGTGTGCTCTTCCGATCT
	P7_B2	CAAGCAGAAGACGGCATACGAGATctcagcGTGACTGGAGTTCAGACGTGTGCTCTTCCGATCT
	P7_B3	CAAGCAGAAGACGGCATACGAGATtccggtGTGACTGGAGTTCAGACGTGTGCTCTTCCGATCT
	P7_B4	CAAGCAGAAGACGGCATACGAGATtgcagtGTGACTGGAGTTCAGACGTGTGCTCTTCCGATCT
	P7_B5	CAAGCAGAAGACGGCATACGAGATttcataGTGACTGGAGTTCAGACGTGTGCTCTTCCGATCT
	P7_B6	CAAGCAGAAGACGGCATACGAGATatacacGTGACTGGAGTTCAGACGTGTGCTCTTCCGATCT
	P7_B7	CAAGCAGAAGACGGCATACGAGATcgttatGTGACTGGAGTTCAGACGTGTGCTCTTCCGATCT

	P7_B8	CAAGCAGAAGACGGCATACGAGATctcggaGTGACTGGAGTTCAGACGTGTGCTCTTCCGATCT
	P7_B9	CAAGCAGAAGACGGCATACGAGATtgtgtgGTGACTGGAGTTCAGACGTGTGCTCTTCCGATCT
	P7_B10	CAAGCAGAAGACGGCATACGAGATaccgcgGTGACTGGAGTTCAGACGTGTGCTCTTCCGATCT
	P7_B11	CAAGCAGAAGACGGCATACGAGATgatcggGTGACTGGAGTTCAGACGTGTGCTCTTCCGATCT
	P7_B12	CAAGCAGAAGACGGCATACGAGATtcacggGTGACTGGAGTTCAGACGTGTGCTCTTCCGATCT
	P7_C1	CAAGCAGAAGACGGCATACGAGATattactGTGACTGGAGTTCAGACGTGTGCTCTTCCGATCT
	P7_C2	CAAGCAGAAGACGGCATACGAGATcttagaGTGACTGGAGTTCAGACGTGTGCTCTTCCGATCT
	P7_C3	CAAGCAGAAGACGGCATACGAGATgcagctGTGACTGGAGTTCAGACGTGTGCTCTTCCGATCT
	P7_C4	CAAGCAGAAGACGGCATACGAGATtcctccGTGACTGGAGTTCAGACGTGTGCTCTTCCGATCT
	P7_C5	CAAGCAGAAGACGGCATACGAGATgaactaGTGACTGGAGTTCAGACGTGTGCTCTTCCGATCT
	P7_C6	CAAGCAGAAGACGGCATACGAGATacaaccGTGACTGGAGTTCAGACGTGTGCTCTTCCGATCT
	P7_C7	CAAGCAGAAGACGGCATACGAGATggtaacGTGACTGGAGTTCAGACGTGTGCTCTTCCGATCT
	P7_C8	CAAGCAGAAGACGGCATACGAGATgtggtcGTGACTGGAGTTCAGACGTGTGCTCTTCCGATCT
	P7_C9	CAAGCAGAAGACGGCATACGAGATccgcgtGTGACTGGAGTTCAGACGTGTGCTCTTCCGATCT
	P7_C10	CAAGCAGAAGACGGCATACGAGATctgacaGTGACTGGAGTTCAGACGTGTGCTCTTCCGATCT
	P7_C11	CAAGCAGAAGACGGCATACGAGATccgaatGTGACTGGAGTTCAGACGTGTGCTCTTCCGATCT
	P7_C12	CAAGCAGAAGACGGCATACGAGATagccgcGTGACTGGAGTTCAGACGTGTGCTCTTCCGATCT
	P7_D1	CAAGCAGAAGACGGCATACGAGATtagcgcGTGACTGGAGTTCAGACGTGTGCTCTTCCGATCT
	P7_D2	CAAGCAGAAGACGGCATACGAGATtgacctGTGACTGGAGTTCAGACGTGTGCTCTTCCGATCT
	P7_D3	CAAGCAGAAGACGGCATACGAGATcttatcGTGACTGGAGTTCAGACGTGTGCTCTTCCGATCT
	P7_D4	CAAGCAGAAGACGGCATACGAGATgtagccGTGACTGGAGTTCAGACGTGTGCTCTTCCGATCT
	P7_D5	CAAGCAGAAGACGGCATACGAGATccatagGTGACTGGAGTTCAGACGTGTGCTCTTCCGATCT
	P7_D6	CAAGCAGAAGACGGCATACGAGATgaggcaGTGACTGGAGTTCAGACGTGTGCTCTTCCGATCT
	P7_D7	CAAGCAGAAGACGGCATACGAGATaattgaGTGACTGGAGTTCAGACGTGTGCTCTTCCGATCT
	P7_D8	CAAGCAGAAGACGGCATACGAGATactcacGTGACTGGAGTTCAGACGTGTGCTCTTCCGATCT
	P7_D9	CAAGCAGAAGACGGCATACGAGATaagttgGTGACTGGAGTTCAGACGTGTGCTCTTCCGATCT
	P7_D10	CAAGCAGAAGACGGCATACGAGATtacgatGTGACTGGAGTTCAGACGTGTGCTCTTCCGATCT
	P7_D11	CAAGCAGAAGACGGCATACGAGATcaccacGTGACTGGAGTTCAGACGTGTGCTCTTCCGATCT
	P7_D12	CAAGCAGAAGACGGCATACGAGATgcattcGTGACTGGAGTTCAGACGTGTGCTCTTCCGATCT
	P7_E1	CAAGCAGAAGACGGCATACGAGATttccatGTGACTGGAGTTCAGACGTGTGCTCTTCCGATCT
	P7_E2	CAAGCAGAAGACGGCATACGAGATaggcgaGTGACTGGAGTTCAGACGTGTGCTCTTCCGATCT
	P7_E3	CAAGCAGAAGACGGCATACGAGATctctcgGTGACTGGAGTTCAGACGTGTGCTCTTCCGATCT
	P7_E4	CAAGCAGAAGACGGCATACGAGATgccttgGTGACTGGAGTTCAGACGTGTGCTCTTCCGATCT

	P7_E5	CAAGCAGAAGACGGCATACGAGATaggttcGTGACTGGAGTTCAGACGTGTG CTCTTCCGATCT
	P7_E6	CAAGCAGAAGACGGCATACGAGATtagcagGTGACTGGAGTTCAGACGTGTG CTCTTCCGATCT
	P7_E7	CAAGCAGAAGACGGCATACGAGATcactgcGTGACTGGAGTTCAGACGTGTG CTCTTCCGATCT
	P7_E8	CAAGCAGAAGACGGCATACGAGATagtggcGTGACTGGAGTTCAGACGTGTG CTCTTCCGATCT
	P7_E9	CAAGCAGAAGACGGCATACGAGATatgtagGTGACTGGAGTTCAGACGTGTG CTCTTCCGATCT
	P7_E10	CAAGCAGAAGACGGCATACGAGATggtacgGTGACTGGAGTTCAGACGTGTG CTCTTCCGATCT
	P7_E11	CAAGCAGAAGACGGCATACGAGATtaaggtGTGACTGGAGTTCAGACGTGTG CTCTTCCGATCT
	P7_E12	CAAGCAGAAGACGGCATACGAGATcctgtaGTGACTGGAGTTCAGACGTGTG CTCTTCCGATCT
	P7_F1	CAAGCAGAAGACGGCATACGAGATcctccaGTGACTGGAGTTCAGACGTGTG CTCTTCCGATCT
	P7_F2	CAAGCAGAAGACGGCATACGAGATacaataGTGACTGGAGTTCAGACGTGTG CTCTTCCGATCT
	P7_F3	CAAGCAGAAGACGGCATACGAGATactgatGTGACTGGAGTTCAGACGTGTG CTCTTCCGATCT
	P7_F4	CAAGCAGAAGACGGCATACGAGATatgtgtGTGACTGGAGTTCAGACGTGTG CTCTTCCGATCT
	P7_F5	CAAGCAGAAGACGGCATACGAGATgtgtccGTGACTGGAGTTCAGACGTGTG CTCTTCCGATCT
	P7_F6	CAAGCAGAAGACGGCATACGAGATtagagtGTGACTGGAGTTCAGACGTGTG CTCTTCCGATCT
	P7_F7	CAAGCAGAAGACGGCATACGAGATgctaagGTGACTGGAGTTCAGACGTGTG CTCTTCCGATCT
	P7_F8	CAAGCAGAAGACGGCATACGAGATtggactGTGACTGGAGTTCAGACGTGTG CTCTTCCGATCT
	P7_F9	CAAGCAGAAGACGGCATACGAGATcacttaGTGACTGGAGTTCAGACGTGTG CTCTTCCGATCT
	P7_F10	CAAGCAGAAGACGGCATACGAGATctattgGTGACTGGAGTTCAGACGTGTG CTCTTCCGATCT
	P7_F11	CAAGCAGAAGACGGCATACGAGATaatacaGTGACTGGAGTTCAGACGTGTG CTCTTCCGATCT
	P7_F12	CAAGCAGAAGACGGCATACGAGATattcttGTGACTGGAGTTCAGACGTGTG CTCTTCCGATCT
	P7_G1	CAAGCAGAAGACGGCATACGAGATggcatcGTGACTGGAGTTCAGACGTGTG CTCTTCCGATCT
	P7_G2	CAAGCAGAAGACGGCATACGAGATggttggGTGACTGGAGTTCAGACGTGTG CTCTTCCGATCT
	P7_G3	CAAGCAGAAGACGGCATACGAGATttggcgGTGACTGGAGTTCAGACGTGTG CTCTTCCGATCT
	P7_G4	CAAGCAGAAGACGGCATACGAGATctgctgGTGACTGGAGTTCAGACGTGTG CTCTTCCGATCT
	P7_G5	CAAGCAGAAGACGGCATACGAGATtgagtaGTGACTGGAGTTCAGACGTGTG CTCTTCCGATCT
	P7_G6	CAAGCAGAAGACGGCATACGAGATctaagtGTGACTGGAGTTCAGACGTGTG CTCTTCCGATCT
	P7_G7	CAAGCAGAAGACGGCATACGAGATagaaggGTGACTGGAGTTCAGACGTGTG CTCTTCCGATCT
	P7_G8	CAAGCAGAAGACGGCATACGAGATtcggacGTGACTGGAGTTCAGACGTGTG CTCTTCCGATCT
	P7_G9	CAAGCAGAAGACGGCATACGAGATtgttcaGTGACTGGAGTTCAGACGTGTG CTCTTCCGATCT
	P7_G10	CAAGCAGAAGACGGCATACGAGATaaccttGTGACTGGAGTTCAGACGTGTG CTCTTCCGATCT
	P7_G11	CAAGCAGAAGACGGCATACGAGATggtcgtGTGACTGGAGTTCAGACGTGTG CTCTTCCGATCT
	P7_G12	CAAGCAGAAGACGGCATACGAGATtatcctGTGACTGGAGTTCAGACGTGTG CTCTTCCGATCT
	P7_H1	CAAGCAGAAGACGGCATACGAGATggcgacGTGACTGGAGTTCAGACGTGTG CTCTTCCGATCT

	P7_H2	CAAGCAGAAGACGGCATACGAGATgtgcctGTGACTGGAGTTCAGACGTGTG CTCTTCCGATCT
	P7_H3	CAAGCAGAAGACGGCATACGAGATgctgccGTGACTGGAGTTCAGACGTGTG CTCTTCCGATCT
	P7_H4	CAAGCAGAAGACGGCATACGAGATccgacgGTGACTGGAGTTCAGACGTGTG CTCTTCCGATCT
	P7_H5	CAAGCAGAAGACGGCATACGAGATaccacaGTGACTGGAGTTCAGACGTGTG CTCTTCCGATCT
	P7_H6	CAAGCAGAAGACGGCATACGAGATctatgaGTGACTGGAGTTCAGACGTGTG CTCTTCCGATCT
	P7_H7	CAAGCAGAAGACGGCATACGAGATtactctGTGACTGGAGTTCAGACGTGTG CTCTTCCGATCT
	P7_H8	CAAGCAGAAGACGGCATACGAGATgacagaGTGACTGGAGTTCAGACGTGTG CTCTTCCGATCT
	P7_H9	CAAGCAGAAGACGGCATACGAGATgcgctaGTGACTGGAGTTCAGACGTGTG CTCTTCCGATCT
	P7_H10	CAAGCAGAAGACGGCATACGAGATgaagtcGTGACTGGAGTTCAGACGTGTG CTCTTCCGATCT
	P7_H11	CAAGCAGAAGACGGCATACGAGATttgattGTGACTGGAGTTCAGACGTGTG CTCTTCCGATCT
	P7_H12	CAAGCAGAAGACGGCATACGAGATcgactcGTGACTGGAGTTCAGACGTGTG CTCTTCCGATCT

SUPPLEMENTAL FIGURES AND LEGENDS

Figure S1. *tolC* is a Dual Selectable Marker in *Escherichia coli*.

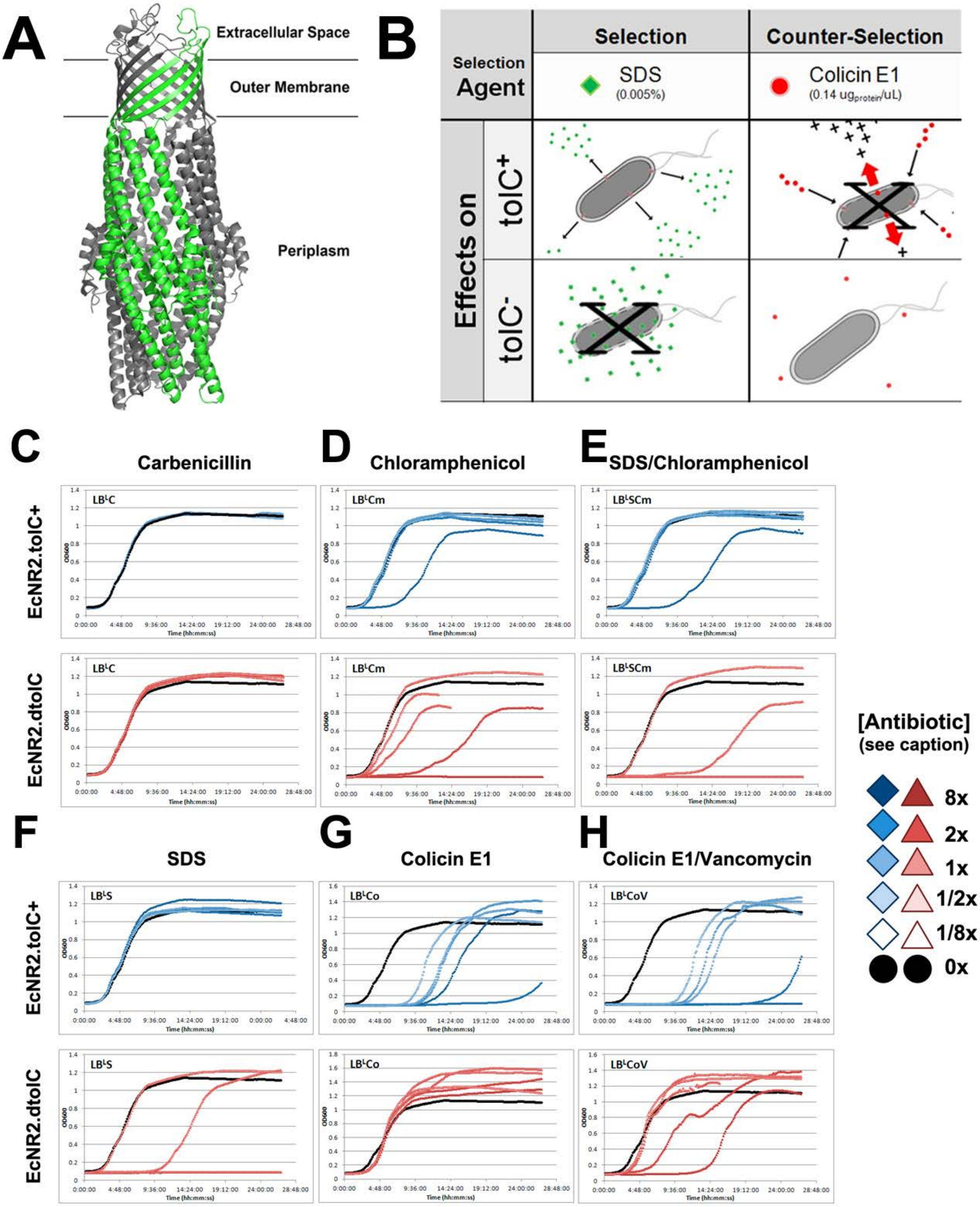

Figure S1. *tolC* is a Dual Selectable Marker in *Escherichia coli*. A. The ribbon structure of *tolC*, based on PDB Entry 1EK9 (17), showing the mature homotrimer. A *tolC* monomer is highlighted in green to show its contribution to the mature protein. The channel is oriented with the extracellular pore on top and the periplasmic pore on bottom with the outer membrane (OM) represented as two black lines. **B.** A table showing the details of *tolC*-based selections. To select for *tolC*-expressing cells, 0.005% sodium dodecyl sulfate (SDS, represented as a green diamond) is used. At this concentration, cells require *tolC* to export SDS to maintain membrane integrity. As such, *tolC* deletion mutants (Δ*tolC*) and cells that have recombined a *tolC*_mut oligo breaking the coding region (*tolC*$^-$) are sensitive to SDS. To select for *tolC*-deficient cells (*tolC*$^-$, Δ*tolC*), 0.14 μg/μL colicin E1 (colE1, represented as a red circle) is used. TolC is required for colE1 to enter the periplasmic space (PP), where colE1 disrupts inner membrane (IM) integrity. As such, *tolC*-expressing cells are sensitive to colE1. **C.** Kinetic growth curves of $5*10^6$ stationary phase EcNR2 (*tolC*$^+$, blue traces, top panel) and EcNR2.ΔtolC (red traces, bottom panel) cells in LB^L + 0x, + 1/8x, + 1/2x, + 1x, + 2x, or + 8x carbenicillin (C), where x = 50 $\mu g_{carbenicillin}$/mL. **D.** As in **C**, except the antibiotic is chloramphenicol (Cm) and x = 20 $\mu g_{chloramphenicol}$/mL. **E.** As in **C**, except that the selective agents are Cm plus sodium dodecylsulfate (SDS) and x_{SDS} = 0.005%. **F.** As in **C**, except that the selective agents are SDS only and x = 0.005%. **G.** As in **C**, except that the selective agents are colicin E1 (colE1) and x = 0.14 $\mu g_{protein}$/uL. **H.** As is **C**, except that the selective agents are colicin E1 and vancomycin (V) and x = 64 $\mu g_{vancomycin}$/mL. This experiment shows the growth phenotype of EcNR2.*tolC*$^+$ and EcNR2.*ΔtolC* in carbenicillin (non-selective), chloramphenicol (selective), SDS (selective), Colicin E1 (counter-selective) and vancomycin (counter-selective). The data are kinetic growth curves monitored for cultures in a 96-well plate format. The x-axis is time (from 0 to 29 hours) and the y-axis is OD_{600}. These data are single, representative replicates from an experiment that has been performed at least three times.

Figure S2. Motivations for CoS-MAGE Cycling.

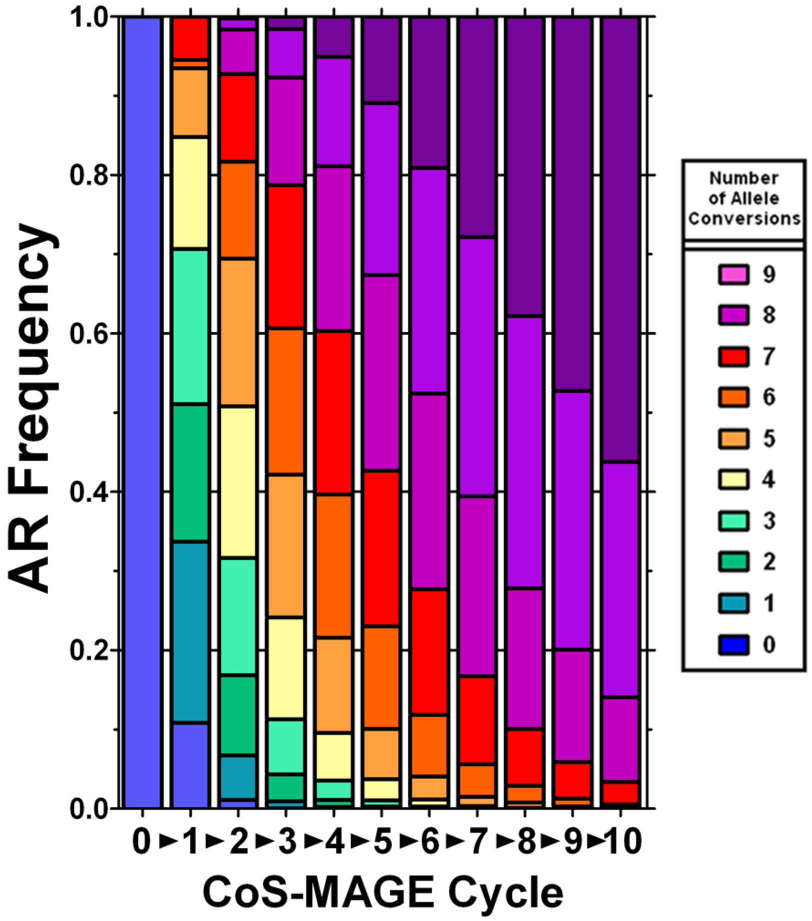

Figure S2. Motivations for CoS-MAGE Cycling. The data from strain EcNR2.Nuc5$^-$.*dnaG*_Q576A (*dnaG*^{Q576A}, Nuc5$^-$, CoS$^+$) were used to model the allele conversion distribution through 10 cycles of CoS-MAGE, to understand how a mixed population will approach the isogenic modified population (e.g., ~100% of cells exhibiting 10/10 of desired mutations). These data were derived from the model that accounted for empirical, positional dependence for conversion of certain pairs of alleles. Both models predict that ~50% of cells will carry 10/10 mutations after 10 cycles of CoS-MAGE, suggesting that CoS-MAGE will need only ~10% of the cycles that MAGE would need to similarly convert the same set of 10 mutations (13). The data are reported as a stacked bar graph where each color indicates the frequency of clones bearing that number of allele conversions.

Figure S3. Repetitive *tolC* Counter-selection Rapidly Generates a Dysfunctional Phenotype.

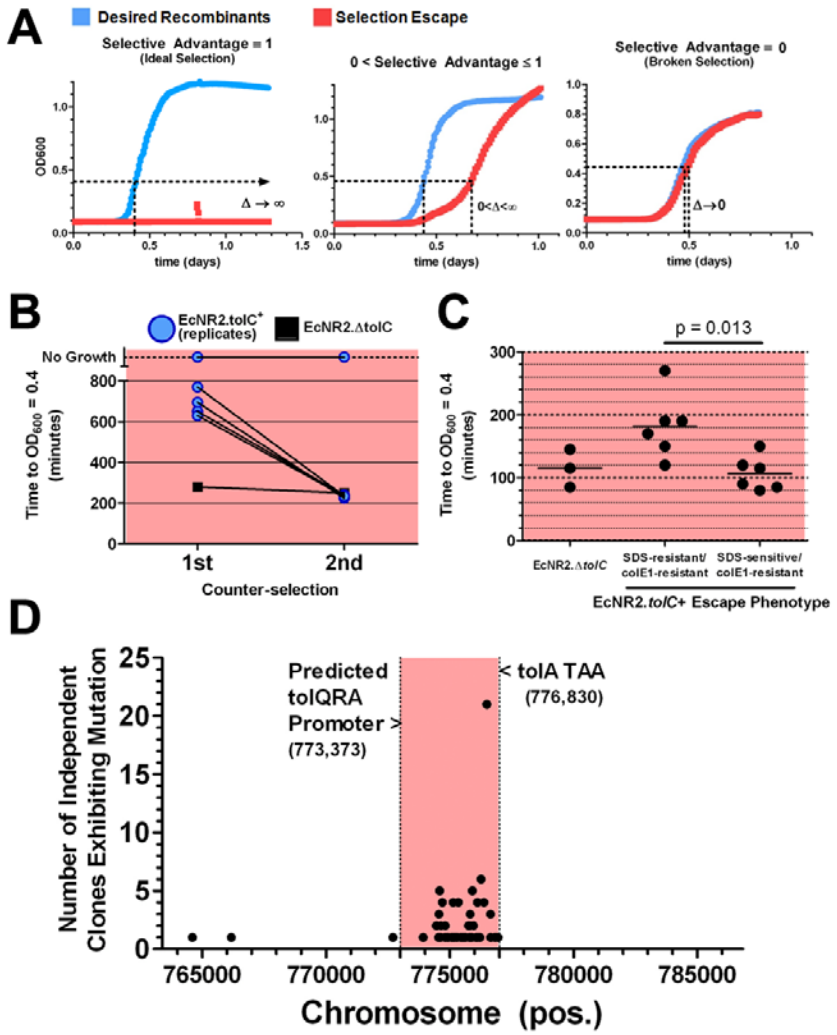

Figure S3. Repetitive *tolC* Counter-selection Rapidly Generates a Dysfunctional Phenotype. A. Theoretical selection kinetics are presented to explain the possible results of typical selections and how we quantified Normalized Selective Advantage (NSA). In all panels, we present the selection kinetics of a population recombined with an oligo to change the state of a selectable marker (e.g., *tolC*$^-$ → *tolC*$^+$, 'CoS-MAGE Recombinants', blue circles), and a control population recombined with water only ('Negative Control', red squares). Thus, the selection kinetics of the negative control is indicative of the background selection escape. At left, we present an ideal selection, where the negative control never grows, defined as Selection Advantage = 1. At center, we present a more commonly-observed selection, in which the desired population (blue circles) exhibits a non-zero growth advantage (Δ), which is the relative advantage that the desired population exhibits over the negative control (red squares). This advantage is translated into a NSA between 0 and 1, using $1- [t^{RS}*(t^{CNS}/t^{RNS})]/t^{CS}$, as discussed in ***tolC*-based Selections** of the **Methods**). At right, we present a broken selection, where the negative control grows at the same rate as the recombinants (NSA = 0). In dysfunctional *tolC* selections, the population is genotypically *tolC*+, but phenotypically SDS-resistant and colE1-resistant. **B.** To test whether counter-selection escape in colE1 is due to loss of colE1 activity over time

or due to genetic escape, we performed biological replicates of colE1 selections on $5*10^5$ naïve EcNR2.*tolC*$^+$. 2/6 selections exhibited no growth (i.e. ideal), whereas the other 4 replicates escaped the 1st Counter-selection. The wells were then passaged directly into colE1 selective media, where the 4 replicates that escaped the 1st counter-selection grew in the 2nd Counter-selection immediately with EcNR2.*ΔtolC* controls (i.e. broken). **C.** We plated escaped cultures from the 1st counter-selection (**B**) onto carbenicillin agar (non-selective media), then replica plated onto SDS agar (selective media). We picked 6 colonies that phenotyped as SDS-resistant/colE1-resistant and 6 that phenotyped as SDS-sensitive/colE1-resistant and measured their growth rates in fresh colE1 media, compared to 3 replicates of EcNR2.Δ*tolC* positive control. These data are presented as the culture time in minutes to reach OD_{600} = 0.4. While SDS-sensitive/colE1-resistant clones grow identically to EcNR2.Δ*tolC* positive controls, SDS-resistant/colE1-resistant clones exhibit a significant delay in growth in colE1 ($p = 0.013$, SDS-resistant/colE1-resistant vs. SDS-sensitive/colE1-resistant using Mann-Whitney test). **D.** After high-throughput sequencing of clones bearing the dysfunctional, *tolC* counter-selection escape phenotype (see Fig. 2), we found enrichment for mutations in the *tolQRA* operon (see Fig. 2A). Here we present a kb-resolution plot of mutations occurring from 763,373 to 786,830, including the *tolQRA* operon from the beginning of the predicted promoter (Chr. 773,373) to the end of the tolA coding region (Chr. 776,830). The *tolQRA* region of interest marked by vertical, dotted lines and a light red background. These data are presented as mutation position on the reference chromosome (x-axis) versus the number of clones (out of 96) which exhibited a mutation at this position (y-axis).

Figure S4. CoS-MAGE Strain Improvements.

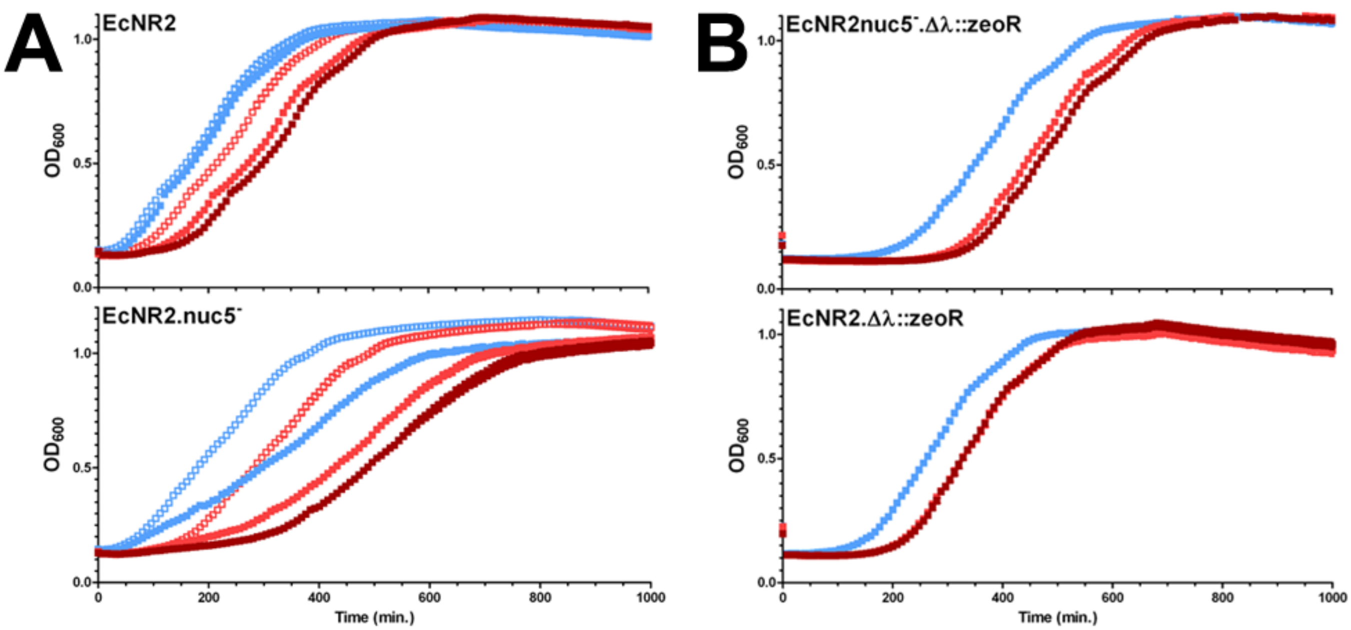

C

Condition Description		V_{max} ($d^2OD_{600}/dt^2 = 0$)			
		E2	E2.nuc5-	E2.dλ:zeoR	E2.nuc5-.dλ:zeoR
Washed	□	6.072	6.036	ND	ND
Washed Electroporated (water)	□	5.976	6.222	ND	ND
Induced Washed	■	8.124	4.056	5.898	6.192
Induced Washed Electroporated (water)	■	6.162	4.35	6.942	6.342
Induced Washed Electroporated (oligo)	■	6.03	3.954	6.336	5.964
Normal Passage		6.702	7.068	7.056	6.366

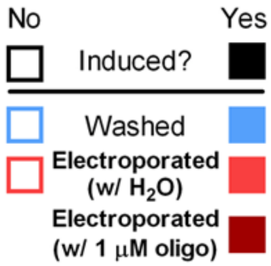

D

Condition Description		V_{max} ($d^2OD_{600}/dt^2 = 0$)					
		E2	E2.nuc5-	E2.nuc5-.exoX+	E2.nuc5-.recJ+	E2.nuc5-.xonA+	E2.nuc5-.xseA+
Induced Washed	□	6.306	5.334	6.432	6.330	3.966	6.696
Induced Washed Electroporated (water)	■	6.606	3.792	5.610	7.686	5.478	6.768
Normal Passage		6.642	5.652	6.630	6.546	5.622	6.594

Figure S4. CoS-MAGE Strain Improvements. To probe the post-recombination phenotype of Nuc5$^-$-based strains, we performed mock recombinations using modified protocols that eliminated certain components of the recombination workflow and allowed us to isolate the effects of each component. The standard recombination protocol includes heat shock-based recombinase induction (“Induced”), icing the culture and performing 2 ice cold water washes (“Washed”, blue squares), and electroporation, either in water (“Electroporated (w/ H_2O)”, red squares) or with a multiplexed oligo pool (“Electroporated (w/ 5.2 μM oligo)”, dark red squares). **A.** Using post-recombination growth as a metric, we compared these conditions using EcNR2 and EcNR2.Nuc5$^-$. Nuc5$^-$ strains exhibit reduced fitness, which is associated with λ Red induction (bottom panel, filled squares). This phenotype was not observed in un-induced EcNR2.Nuc5$^-$ (bottom panel, open squares), nor in nuclease competent strains under any condition (top panel). **B.** To investigate the contribution of λ Red expression during heat shock induction, we generated strains where the λ prophage was replaced by *zeoR* cassette (Δλ::*zeoR*) then repeated **A**. Importantly, we see that EcNR2.Nuc5$^-$ does not exhibit induction-associated

phenotypes when the λ prophage has been deleted, suggesting that the nuclease knockout and induction-associated λ overexpression cause the re-growth phenotype. **C.** Based on the kinetics in **A** and **B**, we report V_{max} ($d^2OD_{600}/dt^2 = 0$) for EcNR2, EcNR2.nuc5$^-$, EcNR2.Δλ::zeoR, EcNR2.nuc5$^-$.Δλ::*zeoR* in the mock recombination conditions tested here. **D.** Based on the kinetics in Fig. 3A, we report V_{max} ($d^2OD_{600}/dt^2 = 0$) for EcNR2, EcNR2.nuc5$^-$, EcNR2.nuc5$^-$.*exoX*$^+$, EcNR2.nuc5$^-$.*recJ*$^+$, EcNR2.nuc5$^-$.*xonA*$^+$, and EcNR2.nuc5$^-$.*xseA*$^+$ in the subset of mock recombination conditions tested there.

Figure S5. Appending the ssrA Degradation Tag to *tolC* Improves Protein Turnover, but Leads to Reduced Fitness in SDS.

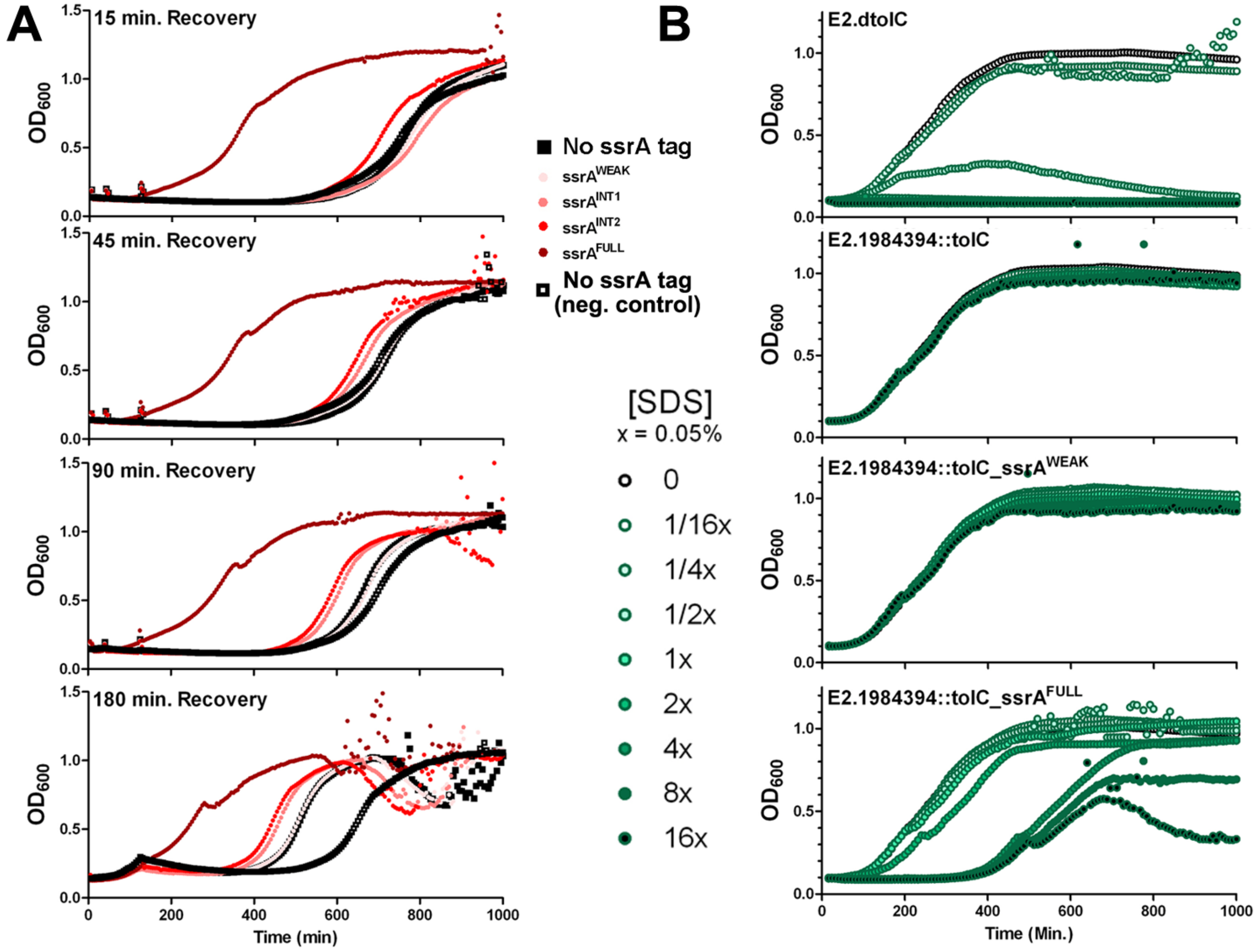

Figure S5. Appending the ssrA Degradation Tag to *tolC* Improves Protein Turnover, but Leads to Reduced Fitness in SDS. We attempted to reduce the need for lengthy recoveries after *tolC* inactivation before counter-selection by optimizing TolC turnover rate using variants of the ssrA tag (6) in frame at the penultimate codon of *tolC*. These variants included ssrA^{full} (AANDENYALAA), ssrA^{int1} (AANDENYALAS), ssrA^{int2} (AANDENYADAA), & ssrA^{weak} (AANDENYADAS). **A.** Recovery time prior to colE1 selection was determined for ssrA-tagged *tolC* strains recombined with *tolC*-r.null_mut (filled shapes) or water (open shapes). We tested EcNR2.1984394::*tolC* (black square), EcNR2.1984394::*tolC*_ssrA^{FULL} (AANDENYALAA, dark red circle), EcNR2.1984394::*tolC*_ssrA^{INT1} (AANDENYALAS, red circle), EcNR2.1984394::*tolC*_ssrA^{INT2} (AANDENYADAA, dark pink circle), and EcNR2.1984394::*tolC*_ssrA^{WEAK} (AANDENYADAS, light pink circle). The recovery time before colE1 selection (from top panel to bottom panel: 15, 45, 90, 180 minutes) is reported in each panel. EcNR2.1984394::*tolC*_ssrA^{FULL} is the only variant that supports normal growth at any time before the normal recovery time of 5 hours. Although ssrA^{INT1} and ssrA^{INT2} yield some advantage over

ssrA^{WEAK} and untagged *tolC* in the 180 minute recovery panel, their growth is not normal and the extra lag time between ssrA^{FULL} and ssrA$^{INT1\ and\ INT2}$ likely reflects incomplete protein turnover and killing of *tolC*$^{-}$/TolC^{+} cells. **B.** Growth in SDS (from 0.00031 w/v or 1/16x to 0.08 w/v or 16x, SDS) of *tolC*_ssrA^{FULL} (EcNR2.1984394::*tolC*_ssrA^{FULL}, bottom panel) and *tolC*_ssrA^{WEAK} (EcNR2.1984394::*tolC*_ssrA^{WEAK}, third panel) was investigated and compared to untagged (EcNR2.1984394::*tolC*, second panel) or *tolC* deletion (EcNR2.Δ*tolC*, top panel) controls. *tolC*_ssrA^{FULL} leads to a significant growth phenotype at 2x SDS and above, whereas untagged *tolC* and *tolC*_ssrA^{WEAK} allow complete SDS tolerance at these concentrations. *tolC* deletion controls show that SDS becomes non-selective at concentrations of ~1/4x; thus, the efficacious concentration window for SDS shrinks from at least 64-fold for untagged *tolC* to ~4-fold for *tolC*_ssrA^{FULL}.

Figure S6. Validating ColE1 Agar Plates to Quantify *tolC* Counter-selection Escape Frequency.

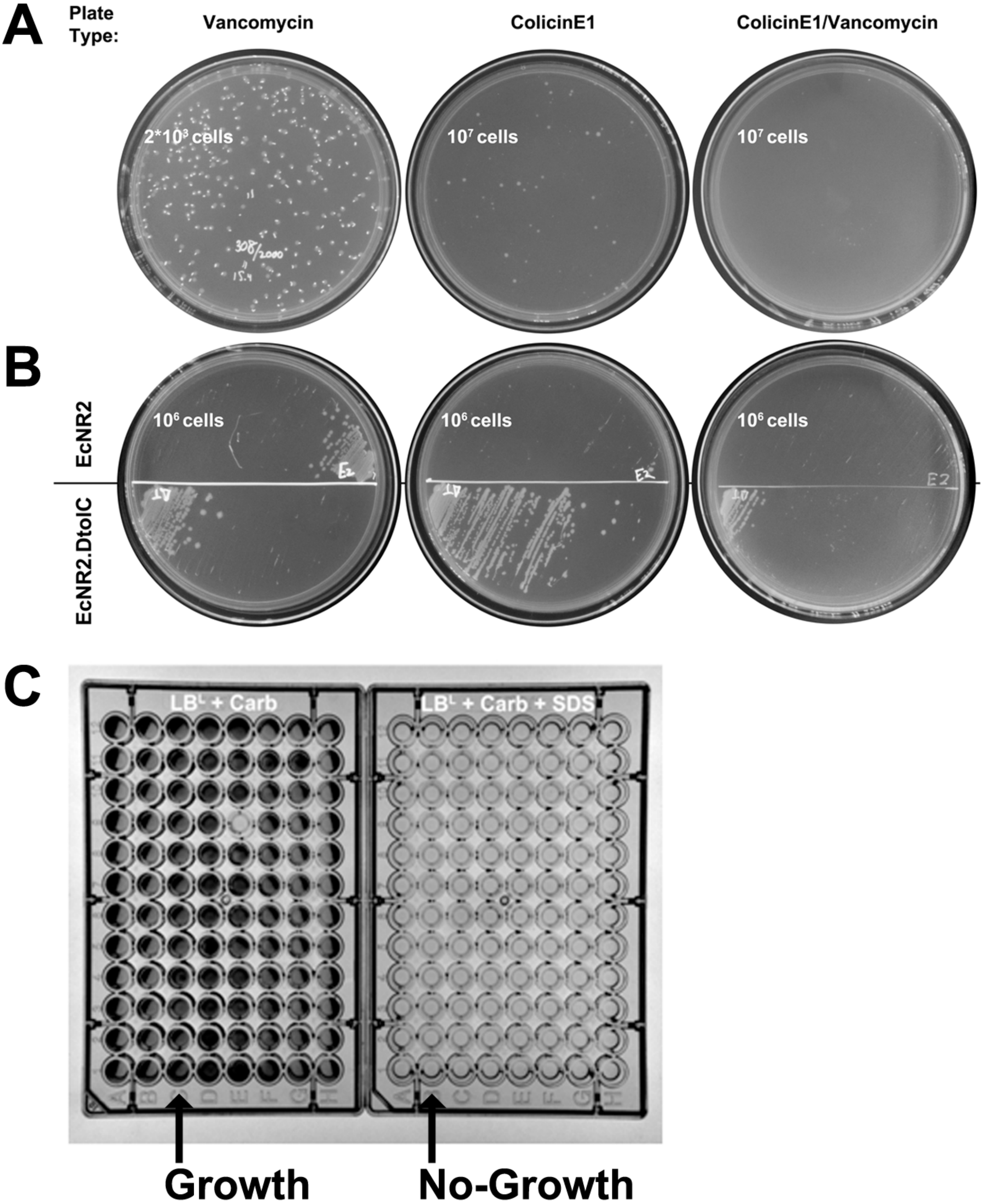

Figure S6. Validating ColE1 Agar Plates to Quantify *tolC* Counter-selection Escape Frequency. A. We made vancomycin (LB^LCV), colE1 (LB^LCCo), and colE1/vancomycin (LB^LCCoV) plates as described in the **Methods**. To validate these plates, we plated $2*10^3$, 10^7 and 10^7 EcNR2.*tolC*$^+$ cells onto LB^LCV, LB^LCCo, and LB^LCCoV plates, respectively. These inocula were chosen for demonstration to yield discrete colonies for counting. Replicates of EcNR2 counter-selection escape frequency (Fig. 4C) are reported here, ordered from least to most robust: LB^LCV, 8.3E-2 ± 1E-1; LB^LCCo, 3.4E-5 ± 1.7E-5; LB^LCCoV, 4.5E-8 ± 9E-9. **B.** We streaked 10^6 EcNR2.*tolC*+ and EcNR2.*ΔtolC* cells onto LB^LCV, LB^LCCo, and LB^LCCoV, respectively, which mirrors the data in **A** showing that the LB^LCCoV plates produce the most striking reduction in counter-selection escape. **C.** We performed a *tolC* inactivating CoS-MAGE cycle on EcM2.0.*tolC*$^+$ using tolC-r.null_mut (0.2 μM) plus a multiplexed oligo pool (5 μM). After recovery, we plated onto LB^LCCoV to perform the *tolC* counter-selection, then picked 96 clones into LB^L supplemented with Carb and LB^L supplemented with Carb and SDS to test if the plates efficiently kill the parental genotype. Growth (dark wells) was seen for 95/96 wells in LB^L supplemented with Carb, whereas none (light wells) grew in LB^L supplemented with Carb and SDS, demonstrating that the plates efficiently kill tolC$^+$ strains and can therefore be used to directly isolate monoclonal *tolC*$^-$ clones.
